# Supplementary material for: RNA binding protein PRRC2B mediates translation of specific mRNAs and regulates cell cycle progression
Source: Nucleic Acids Res. 2023 May 1;51(11):5831–46. doi: 10.1093/nar/gkad322 (PMC10287950; doi:10.1093/nar/gkad322)
Supplement: gkad322_Supplemental_Files [file gkad322_supplemental_files.zip › PRRC2B_Supplemental Information_revised.pdf]

Supplementary Information for

RNA binding protein PRRC2B mediates translation of specific mRNAs and regulates cell cycle progression

Feng Jiang, Omar M. Hedaya, EngSoon Khor, Jiangbin Wu, Matthew Auguste, Peng Yao

Correspondence to Peng Yao

E-mail: peng_yao@urmc.rochester.edu

This document file includes the following:

Figures S1 to S6

Tables S1 to S8

Extended Data 1 and 2

SI Materials and Methods

MIQE for RT-qPCR

Supplemental figures

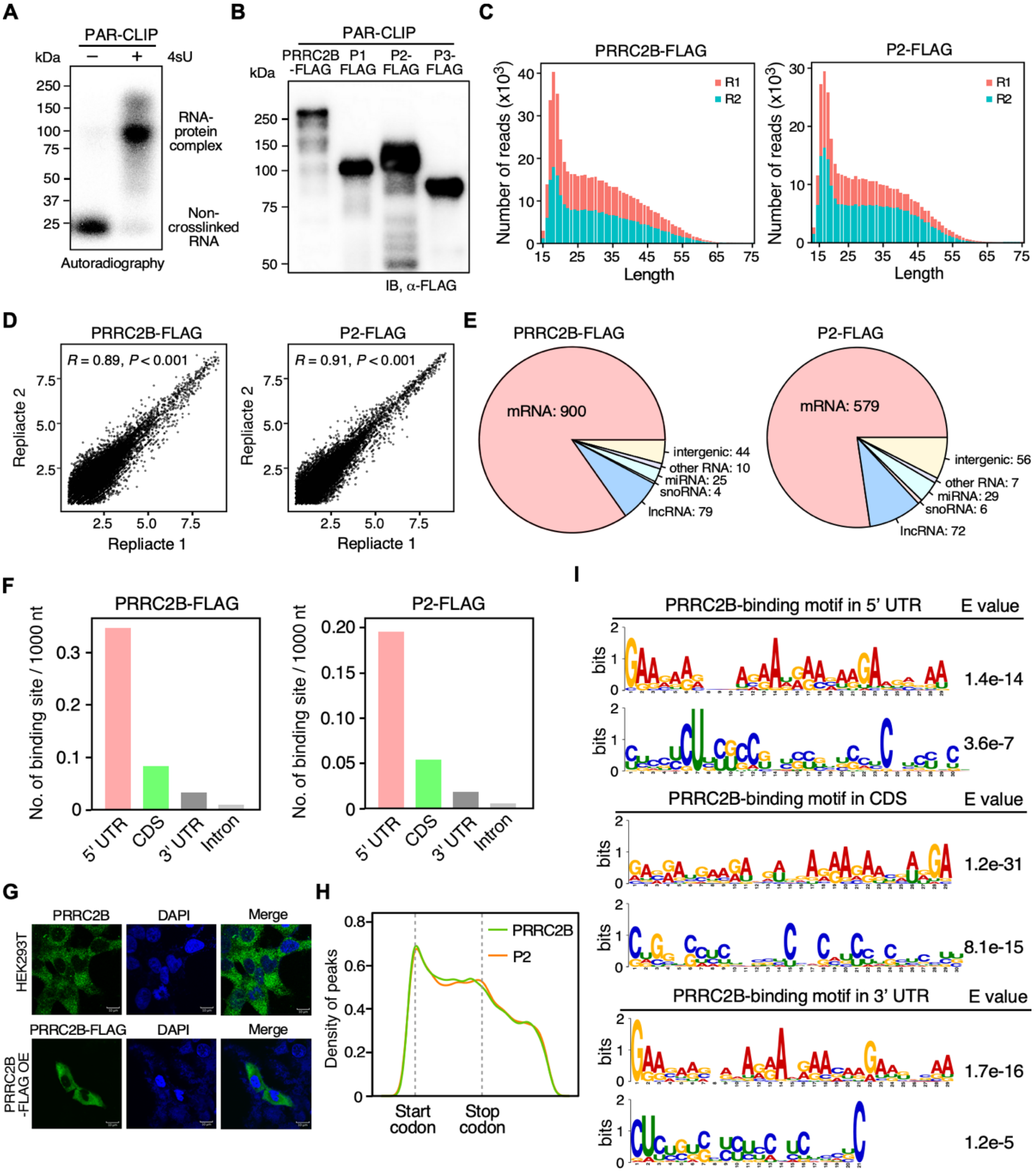

Figure S1. PAR-CLIP analysis of PRRC2B-RNA interaction. (**A**) An autoradiographic image showing the migration of ^{32}[P]-radiolabeled crosslinked RNA-protein complexes (with 4-thiouridine, 4sU) and non-crosslinked RNAs (without 4sU) enriched in the immunoprecipitation step of PAR-CLIP on a 4-12% Bis-Tris gel. (**B**) Western blot analysis

shows the enrichment of comparable protein amounts of FLAG-tagged full-length PRRC2B and fragments (P1, P2, P3) by anti-FLAG antibody-conjugated magnetic beads in PAR-CLIP. 2% of the total volume of enriched proteins was loaded on a 10% SDS-PAGE gel. (**C**) Distribution of the length of sequenced reads after trimming and quality filtering of the two biological replicates in full-length PRRC2B (left) and P2 PAR-CLIP (right). (**D**) Strong correlations (Pearson's correlation) between the two biological replicates in full-length PRRC2B (left) and P2 PAR-CLIP (right), as indicated by scatterplots. The coordinates of each dot represent the FPKM (fragments per kilobase of exon per million mapped fragments) values of each gene in the two biological replicates. (**E**) Distribution of the binding sites identified in full-length PRRC2B (left) and P2 PAR-CLIP (right) on different RNA species and intergenic regions. Binding sites on exons and introns were included. (**F**) Histograms showing the density of binding sites (number of binding sites per 1000 nt) on 5' UTR, CDS, 3' UTR, and intron regions of mRNA in full-length PRRC2B (left) and P2 PAR-CLIP (right). Only the binding sites on mRNAs were considered. (**G**) Subcellular localization of endogenous and FLAG-tagged PRRC2B in HEK293T cells detected by immunofluorescent staining. (**H**) Distribution of binding sites identified in full-length PRRC2B (left) and P2 PAR-CLIP (right) on mature mRNAs. Only the longest transcript was included for each gene. All transcripts were scaled to the same length with a start and stop codons of the main open reading frames aligned. Kernel density estimation (KDE) was plotted as Y-axis. (**I**) The longest significant consensus motifs that MEME can identify from the sequences flanking the T-to-C mutation sites (-20 to +20 nt) in the overlapped binding sites on 5' UTR, CDS, and 3' UTR. Significance was tested against randomized sequences according to the MEME user guide. E-value estimates the expected number of motifs found in a similarly sized set of random sequences (32). E-value < 0.05 was considered statistically significant.

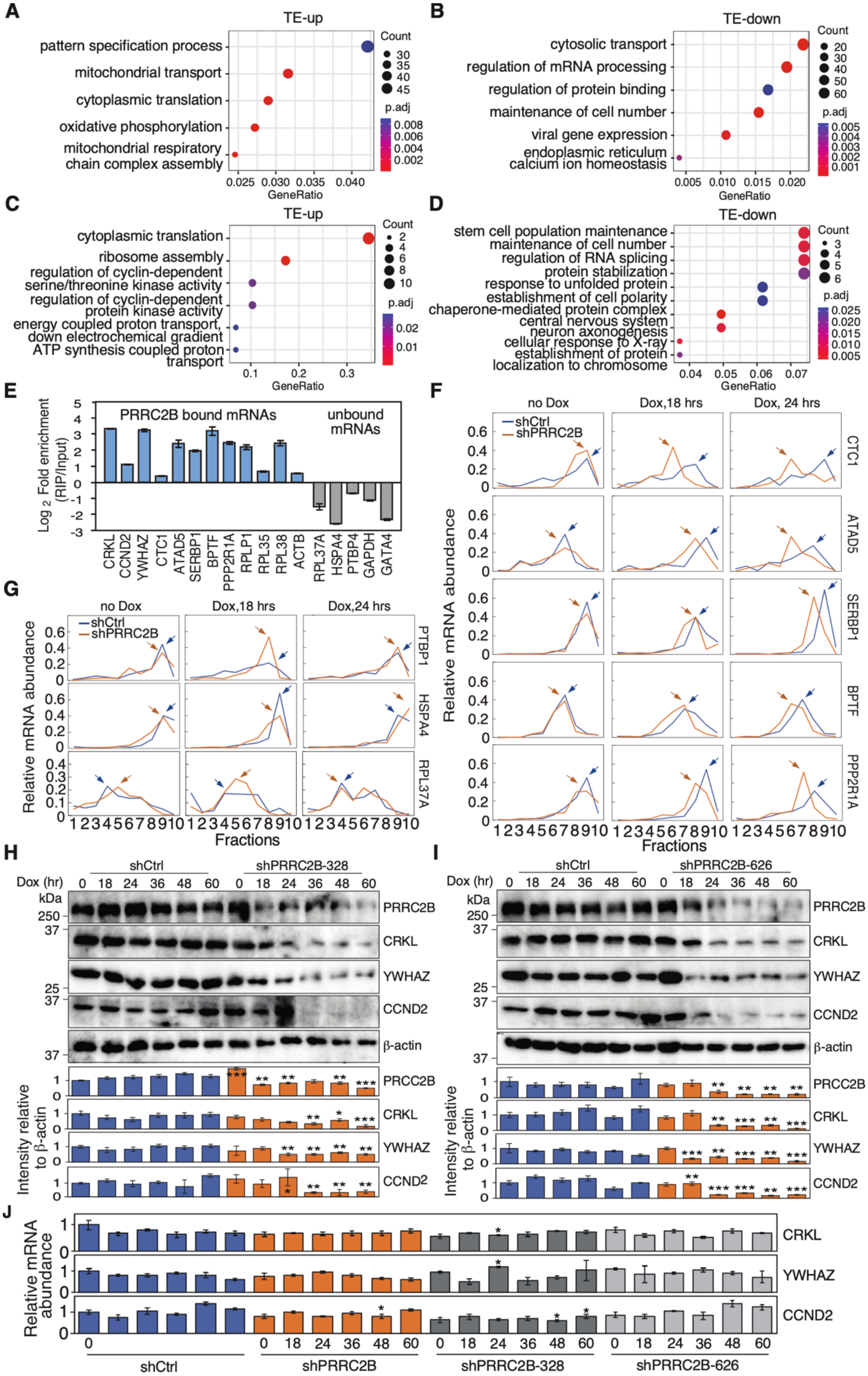
A
TE-up
pattern specification process
mitochondrial transport
cytoplasmic translation
oxidative phosphorylation
mitochondrial respiratory chain complex assembly
GeneRatio
Count
p.adj
B
TE-down
cytosolic transport
regulation of mRNA processing
regulation of protein binding
maintenance of cell number
viral gene expression
endoplasmic reticulum calcium ion homeostasis
C
TE-up
cytoplasmic translation
ribosome assembly
regulation of cyclin-dependent serine/threonine kinase activity
regulation of cyclin-dependent protein kinase activity
energy coupled proton transport, down electrochemical gradient
ATP synthesis coupled proton transport
D
TE-down
stem cell population maintenance
maintenance of cell number
regulation of RNA splicing
protein stabilization
response to unfolded protein
establishment of cell polarity
chaperone-mediated protein complex
central nervous system neuron axonogenesis
cellular response to X-ray
establishment of protein localization to chromosome
E
Log 2 Fold enrichment (RIP/Input)
PRRC2B bound mRNAs
unbound mRNAs
CRKL CCND2 YWHAZ CTC1 ATAD5 SERBP1 BPTF PPP2R1A RPLP1 RPL35 RPL38 ACTB RPL37A HSPA4 PTBP4 GAPDH GATA4
F
no Dox
Dox,18 hrs
Dox, 24 hrs
shCtrl
shPRRC2B
CTC1
ATAD5
SERBP1
BPTF
PPP2R1A
Relative mRNA abundance
Fractions
G
no Dox
Dox,18 hrs
Dox,24 hrs
PTBP1
HSPA4
RPL37A
Relative mRNA abundance
Fractions
H
shCtrl
shPRRC2B-328
Dox (hr) 0 18 24 36 48 60 0 18 24 36 48 60
kDa
PRRC2B
CRKL
YWHAZ
CCND2
β-actin
Intensity relative to β-actin
PRCC2B
I
shCtrl
shPRRC2B-626
J
CRKL
YWHAZ
CCND2
Relative mRNA abundance
shCtrl
shPRRC2B
shPRRC2B-328
shPRRC2B-626

Figure S2. PRRC2B-bound mRNAs exhibit decreased translation efficiencies upon PRRC2B knockdown. (**A-D**) Top enriched gene ontology (GO) biological processes in all TE-up mRNAs, TE-down mRNAs, TE-up PRRC2B-bound mRNAs, and TE-down PRRC2B-bound mRNAs. GO terms were filtered by adjusted $P < 0.05$ and simplified by the R package 'ClusterProfiler' to remove redundant terms. (**E**) RNA-binding protein immunoprecipitation (RIP) of endogenous PRRC2B followed by RT-qPCR for PRRC2B-bound and unbound mRNAs. Input mRNA was used as a normalizer. Results are presented as mean $\pm$ SD. (**F, G**) mRNA abundance distribution (detected by RT-qPCR and represented as a percentage of total RNA) of exemplary TE-down PRRC2B-bound mRNAs (*CTC1*, *ATAD5*, *SERBP1*, *BPTF*, *PPP2R1A*), TE-down unbound mRNAs (*PTBP1*, *HSPA4*), and TE-up unbound mRNAs (*RPL37A*) in different polysome profiling fractions at 0, 18, 24 hours after Dox induction of PRRC2B knockdown. Arrows indicate the peak of mRNA abundance. Biological duplicates were performed for RT-qPCR experiments included in (F, G), and representative data were shown. (**H, I**) Western blot results of the protein abundance of TE-down PRRC2B-bound mRNAs (*CRKL*, *YWHAZ*, *CCND2*) at 0, 18, and 24 hours after Dox induction of PRRC2B knockdown by two additional shRNAs. (**J**) RT-qPCR measurement of *CRKL*, *YWHAZ*, *CCND2* mRNA in control and PRRC2B knockdown cells at different time points after Dox induction. All western blot results are presented with quantification plots and analyzed by the Student's *t* test. Significance was calculated by comparing shPRRC2B to shCtrl at each time point. * $P < 0.05$; ** $P < 0.01$; *** $P < 0.001$.

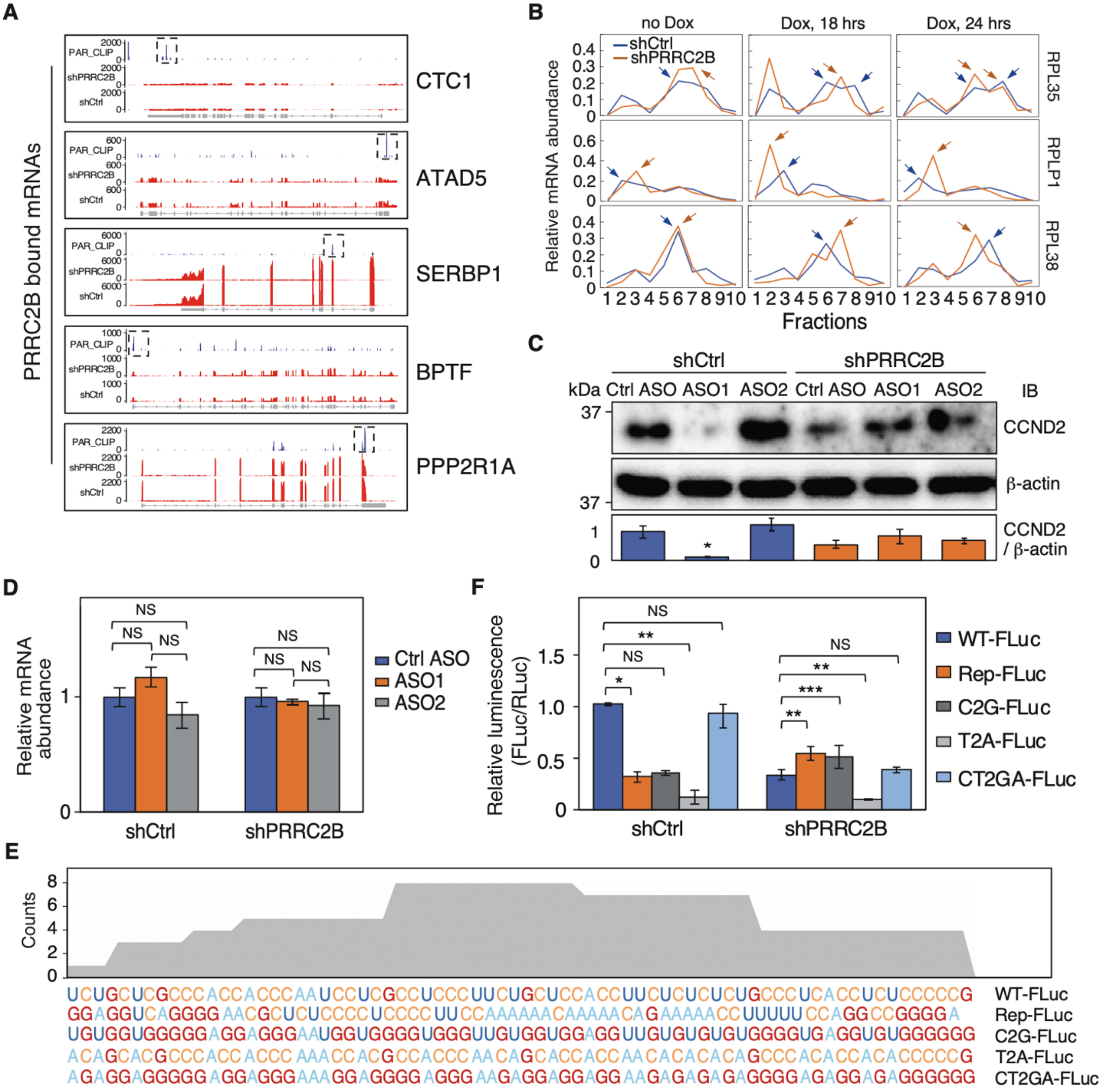

Figure S3. PRRC2B knockdown cells are insensitive to ASOs and express the mutant luciferase reporters differently from wild-type cells. (**A**) Gene body coverages (exhibited as FPKM values) of exemplary TE-down PRRC2B-bound mRNAs (*CTC1*, *ATAD5*, *SERBP1*, *BPTF*, *PPP2R1A*) in PAR-CLIP of full-length PRRC2B, total RNA-seq of control cells, and total RNA-seq of PRRC2B knockdown cells. RPKM values are plotted as Y-axis. (**B**) mRNA abundance distribution (detected by RT-qPCR and represented as a percentage of total RNA) of exemplary TE-up PRRC2B-bound mRNAs in different polysome profiling fractions at 0, 18, and 24 hours after Dox induction of PRRC2B

knockdown. Arrows indicate the peak of mRNA abundance. Biological duplicates were performed, and representative data were shown. (**C**) Western blot analysis of the protein abundance of CCND2 in shCtrl and shPRRC2B cells treated with Ctrl ASO, ASO1, and ASO2. (**D**) RT-qPCR measurement of *CCND2* mRNA in shCtrl and shPRRC2B cells treated with Ctrl ASO, ASO1, and ASO2. (**E**) A snapshot showing the sequences of the PRRC2B binding region on WT-FLuc and corresponding mutated sequences in Del-FLuc, C2G-FLuc, T2A-FLuc, and CT2GA-FLuc. (**F**) Relative luminescence (FLuc/RLuc) of wild-type and four mutant reporters in shCtrl and shPRRC2B cells. To compare the luciferase translation between shCtrl and shPRRC2B, relative luminescence (FLuc/RLuc) was normalized against mRNA abundance (*FLuc*/*RLuc*) and presented as Y-axis values. For all assays involving PRRC2B knockdown, experiments were performed 36 hours after DOX induction. Western blots were representative of >2 biological replicates. Data were expressed as mean ± SD in (C, D, F), and the Student's *t* test was used to calculate the significance. NS, not significant; * $P < 0.05$; ** $P < 0.01$. All western blot results are presented with quantification plots and analyzed by the Student's *t* test. Significance was calculated separately in shCtrl and shPRRC2B cells by comparing ASO1 and ASO2 to Ctrl ASO in (C, D). * $P < 0.05$; ** $P < 0.01$; *** $P < 0.001$.

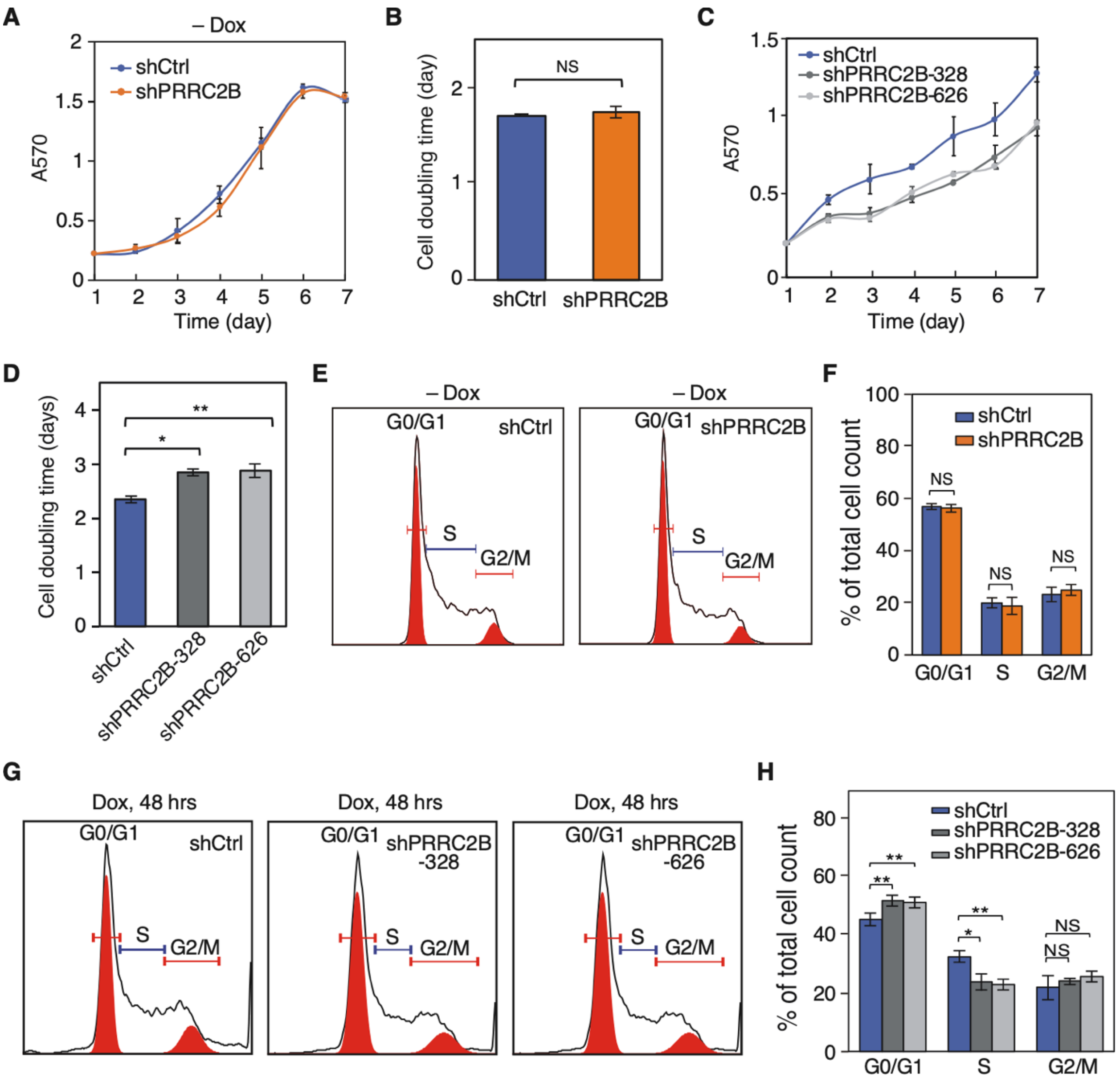

Figure S4. Cell proliferation or G1/S transition does not change without induction of PRRC2B knockdown. (**A**) Cell proliferation curves from day 1 to day 7 of control cells (shCtrl) and PRRC2B knockdown cells (shPRRC2B) without induction of PRRC2B knockdown (no DOX treatment) measured by MTT assay. (**B**) Doubling time was calculated by fitting the cell proliferation data (A) to exponential functions. (**C**) Cell proliferation curves from day 1 to day 7 of control cells (shCtrl) and PRRC2B knockdown cells (shPRRC2B-328, shPRRC2B-626) with induction of PRRC2B knockdown (DOX treatment) measured by MTT assay. (**D**) Doubling time was calculated by fitting the cell proliferation data (C) to exponential functions. (**E**) Representative flow cytometry images

of control (left) and PRRC2B knockdown cells (right) without DOX induction. (**F**) The quantification of relative cell number across different cell cycle stages in (E). (**G**) Representative flow cytometry images of control (shCtrl) and PRRC2B knockdown cells (shPRRC2B-328, shPRRC2B-626) 48 hours after DOX induction. (**H**) The quantification of relative cell number across different cell cycle stages in (G). For all the line plots and histograms, biological triplicates were performed. For all flow cytometry results, biological triplicates were performed, G0/G1, S, and G2/M peaks were autodetected and quantified by FlowJo, and percentages of total cells detected by flow cytometry were reported. Data were expressed as mean $\pm$ SD, and the Student's *t* test was used to calculate the significance. NS, not significant; * $P < 0.05$; ** $P < 0.01$; *** $P < 0.001$.

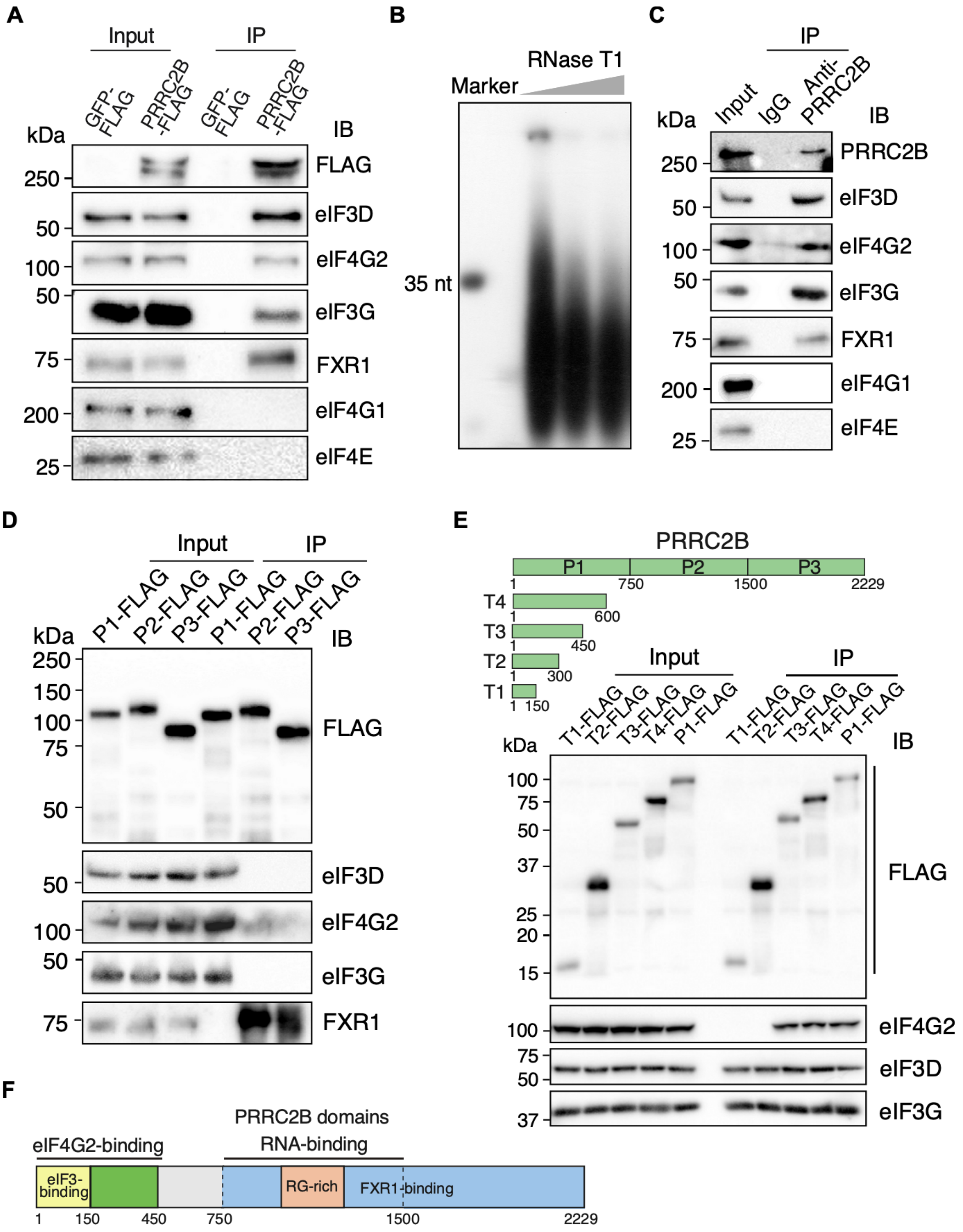

Figure S5. Co-immunoprecipitation of PRRC2B with translation initiation factors. (**A**) Western blot detection of the co-immunoprecipitation (Co-IP) of FLAG-tagged

PRRC2B together with translation initiation factors (eIF4G2 and eIF3) and FXR1 without RNase T1 treatment. (**B**) SYBR gold staining shows the fragmentation of RNA in total cell lysates by 1 U/μl, 5 U/μl, and 10 U/μl RNase T1. (**C**) Western blot detection of the Co-IP of endogenous PRRC2B together with translation initiation factors and FXR1 upon 5 U/μl RNase T1 treatment. (**D**) Western blot detection of the Co-IP of FLAG-tagged PRRC2B fragments (P1, P2, P3) together with translation initiation factors and FXR1 upon 5 U/μl RNase T1 treatment. (**E**) Upper: Schematic of the fragmentation of the P1 region of PRRC2B protein. Truncated P1 (T1-T5) was made by removing a series of 150 amino acids from the C-terminus of P1. Lower: Western blot detection of the Co-IP of FLAG-tagged truncated P1 fragments (T1-T5) together with translation initiation factors upon 5U/μl RNase T1 treatment. (**F**) Schematic of the regions of PRRC2B protein interacting with eIF3, eIF4G2, FXR1, and RNA. Western blots were representative of biological replicates. Western blot results are representative of >2 independent experiments in (A, C, D, E).

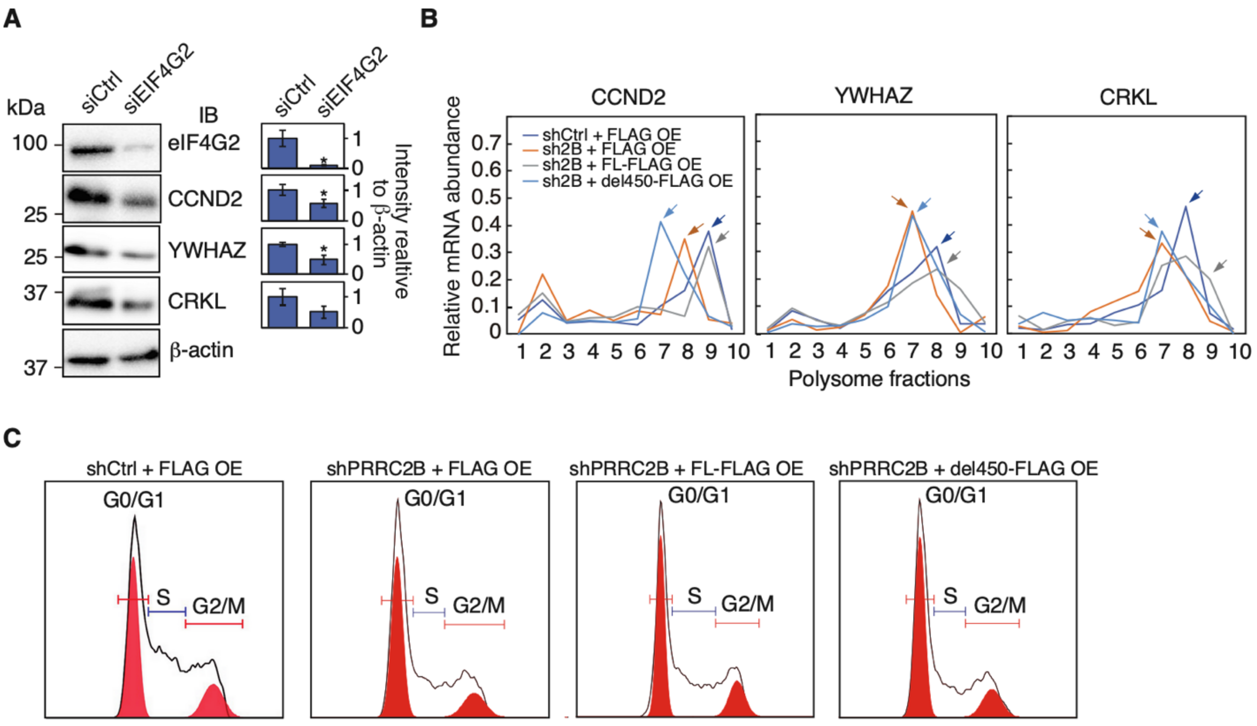

Figure S6. eIF4G2/eIF3-PRRC2B interaction is essential for translation of PRRC2B mRNA targets. (**A**) Western blot detection of the abundance changes of proteins (CCND2, YWHAZ, CRKL) in cells transfected with control siRNA with no target in human cells (siCtrl) and cells transfected with siRNA targeting CDS of *EIF4G2* mRNA (siEIF4G2). (**B**) mRNA abundance distribution (detected by RT-qPCR and represented as a percentage of total RNA) of PRRC2B-bound mRNAs (*CCND2*, *YWHAZ*, *CRKL*) in different polysome profiling fractions from cells under different conditions. Arrows indicate the peak of mRNA abundance. Biological duplicates were performed, and representative data were shown. (**C**) Representative flow cytometry images of cells under different conditions. For all flow cytometry results, biological triplicates were performed. β-actin is used as an internal control for western blot. For all assays involving PRRC2B knockdown, experiments were performed 36 hours after induction of PRRC2B knockdown. Western blots were representative of >2 biological replicates in (A). All quantitative western blot results are presented with quantification plots and analyzed by Student's *t* test. Significance was calculated by comparing to siCtrl in (A). * $P < 0.05$; ** $P < 0.01$; *** $P < 0.001$.

Extended Data 1.

Sequence alignment of PRRC2B protein in five representative species was shown. PRRC2B gene is conserved in 460 species, including all kinds of mammals, reptiles, amphibians, and fish (e.g., zebrafish and shark) based on sequence information from NCBI.

```
zebrafish       MSDRLGQITKSKDGKSKYSSLSLFDKYKGKSIETQKTTVVARHGLQSLGKVAAARRMPPP    60
frog            MSDRLGQITKGKDGKSKYSSLNLFDKYKGKSIEAVRTTVIPRHGLQSLGKVAAARRMPPP    60
chicken         MSDRLGQITKGKDGKSKYSTLSLFDKYKGKSIEAIRTTVIPRHGLQSLGKVAAARRMPPP    60
human           MSDRLGQITKGKDGKSKYSTLSLFDKYKGKSVDAIRSSVIPRHGLQSLGKVAAARRMPPP    60
mouse           MSDRLGQITQGKDGKSKYSTLSLFDKYKGRSVGAVRSSVIPRHGLQSLGKVATARRMPPP    60
                *********:.********:*.*******:*: : :::*: ***********:*******

zebrafish       AHLPSLKSESKGNDPNVIIVPKDGTGWANKQDQPDPKSSVASSAQLPEPQPPLALQKSVS    120
frog            ANLPSLKSENKGNDPNIFIVPKDGTGWANKQEQQDQKSSSVTAPPQQESLPPQGLQKSVS    120
chicken         ANLPSLKSENKGNDPNIVIVPKDGTGWANKQDQSDQKNSSATAAQLQESLLQQGLQKSVS    120
human           ANLPSLKSENKGNDPNIVIVPKDGTGWANKQDQQDPKSSSATASQPPESLPQPGLQKSVS    120
mouse           ANLPSLKSENKGNDPNIVIVPKDGTGWANKQDQQDPKSSSVTASQPPESQPQPGLQKSVS    120
                *:*******.******:.*************:* * *.* .::    *     .******

zebrafish       NLQKPMPITSPEST--STGGPKQWAQLNGKAVDQD-GLKVSSRLQPFSHEDFPTLKAAGE    177
frog            NLQKPTPSINQENINLVPGGPKSWAQLNGKPAGQEGGSRGSNRLLSFSPEEFPTLKAAGE    180
chicken         NLQKPTQSIAQESTNSVPGGPKSWAQLNGKPAGLEGGSRASSRLLSFSPEEFPTLKAAGE    180
human           NLQKPTQSISQENTNSVPGGPKSWAQLNGKPVGHEGGLRGSSRLLSFSPEEFPTLKAAGG    180
mouse           NLQKPTQSISQENTNSVPGGPKSWAQLSGKPVGHEGGLRGSSRLLSFSPEEFPTLKAAGG    180
                *****      *.     ****.****.** .. : * : *.**  ** *:********

zebrafish       QDKVGKERSVFDPSYGPGPSLRPQ--------NVSSWREGGGRNLQPPSLSSAPPGDTDT    229
frog            QDKAGKEKSVLDPSYGPGPSLRPQSKPLALLPDVTSWRDGGGRSIASSLSPTASPTEPGS    240
chicken         QDKVGKEKGALDLSYGPGPSLRPQ--------NVTSWREGGGRNITSATSLTASPAELGS    232
human           QDKAGKEKGVLDLSYGPGPSLRPQ--------NVTSWREGGGRHIISATSLSTSPTELGS    232
mouse           QDKAGKEKGALDLSYGPGPSLRPQ--------NVTSWREGGGRNIISAASLSASPTELGS    232
                ***.***:..:* ***********        :*:***:**** :      :: * : .:

zebrafish       KSSGSVEAPLPPPPSTSTSTSSSASPAASSSSTAAAAAPSSEVKEPSLRPAPPLTRRAAP    289
frog            KTAV-------------------------PGEGPPSTSLANDTKELSLRPAQPTRKGPAQ    275
chicken         KTSS-------------------------TGDGAPSSVSASDPKEPSLRPAQPVRKGASQ    267
human           RNSS-------------------------TGDGAPSSACTSDSKDPSLRPAQPVRKGASQ    267
mouse           RNAS-------------------------GADGAPSLACTSDSKEPSLRPAQPSRRGASQ    267
                :.:                           ..   :   :.: *: ***** *  :  :

zebrafish       PALQYQHHTTTTYHDMLPAFMCPKETREAPGSSDGPAAVVA--PVRFDSRPIFRPPFPAP    347
frog            --FMGNTYHPPTYHDMLPAFMCSQHPPEPSGTLDRTSFPTLNSQSRLEPRVPFRQ-----    328
chicken         --FMGNVYQPPTYHDMLPAFMCPQQPSETPAPLDRGSFPVP--QLRLEPRVPFRQ-----    318
human           --FMGNVYHPPTYHDMLPAFMCSPKSSENQGTVERGSFPLP--QLRLEPRVPFRQ-----    318
mouse           --FMGHGYQPPTYHDMLPAFMCSPQSSENQTTVERSSFPLP--QLRLEPRVPFRQ-----    318
                  :  : :   ***********  .  *      :  :        *:: *  **

zebrafish       EPVNGDVRREPRFPRAPPRPSSRPIRRPGDRAPRPAIINPDDLKDLDELDNDCEDGWAGL    407
frog            FQMNDNEGRESRFPIAPPRS-ARPPRPPAERPPRATIINAEDLKELDDLDNDAEDGWAGL    387
chicken         YQMNDQDGKENRLGLSR--P-ARPLRQLVERAPRPTIINAENLKGLDELDNDADDGWAGI    375
human           FQMNDQDGKENRLGLSR--P-LRPLRQLVERAPRPTIINAENLKGLDDLDADADDGWAGL    375
mouse           FQMNDQDGKE-RPGVAR--P-VRPLRQLVERAPRPTIINAENLKGLDDLDTDADDGWAGL    374
                  :*.:  :* *   :      ** *   :* ** :*** ::** **:** *.:*****:
```

```
zebrafish       HEEVDYSEKLKFSDDEEDHAP--REKSKIWDDWDHHHHHHHLQGQHSSQSSGDGAFPQEP      465
frog            HDEVDYSEKLKFSEDEEEEDGAAKDGRSKWNGWDGKRQRQ---VSFNSTDSVEGKHPADE      444
chicken         HDEVDYSEKLKFSEDEEEEE-SLKDGRQKWNSWDPRRQRQ---LSMSSADSADVKHTLEE      431
human           HEEVDYSEKLKFSDDEEEEE-VVKDGRPKWNSWDPRRQRQ---LSMSSADSADAKRTREE      431
mouse           HEEVDYSEKLKFSDDEDEED-VVKDGRSKWNNWDPRRQRA---LSLSSADSTDAKRTQEE      430
                *:***********:**::.    ::    *:.** ::::     . .* .* :     :

zebrafish       ED--------EPYLR-QQE--PPASRKTSTRFPAEPQQK-----ASVNSESAEEQDEPQR      509
frog            GKVWNDTLSHSRPARRTPEPAPQGPRKPNTWAAVADHQKPPAPAAVLRQPSI---EEKDE      501
chicken         GKNWSDSVGLSRSVRKAQDSQQ-PPRKINGWNSTSEYQKPS-QGSALRQQSL---EDKEE      486
human           GKDWAEAVGASRVVRKAPDPQP-PPRKLHGWAPGPDYQKSS-MGSMFRQQSI---EDKED      486
mouse           GKDWSGTAGGSRVIRKVPEPQP-PSRKLHSWASGPDYQKPT-MGSMFRQHSA---EDKED      485
                 .        .   *   :      **          **     : ... *    :: :

zebrafish       SAPPRGKFVSADV-SAVERARRRREEEERRAREERLAACAEKLKKLDEKFGKTEKPARSG      568
frog            KLPQRKNFVQSEISEAVERARKRREEEERRNREERLAACAAKLKQLDQKNKQALKPSADS      561
chicken         KVPLRQKFVHSEISEAVERARKRREEEERRAREERLAACAAKLKQLDQKCKLAQKSGE--      544
human           KPPPRQKFIQSEMSEAVERARKRREEEERRAREERLAACAAKLKQLDQKCKQARKAGE--      544
mouse           KPPPRQKFIQSEMSEAVERARKRREEEERRAREERLAACAAKLKQLDQKCRQAQKANE--      543
                . * * :*: ::: .******:******** ********* ***:**:*   : *

zebrafish       ----EADGKELAQSPARRSAKPQQDGWQYGSKEV----T--------DTPAESASSQDYK      612
frog            SRQLDNKENEDPRSPVSDRSNPQENAHTFRREYS----QESPSEYPEEEPSGT-------      610
chicken         --TQKHTENEDLRPPNTEKSAVQENGHVFRRATPEFHTQDVSGYLEEETPAPAAP-----      597
human           --ARKQAEKEVPWSPSAEKASPQENGPAVHKGSPEFPAQETPTTFPEEAPTVSPAV-AQS      601
mouse           --TPKPVEKEVPRSPGIEKVSPPENGPVVRKGSPEFPVQEAPTMFLEETPATSPTV-AQS      600
                    .   :*    *        ::.                     : *: :

zebrafish       DEGCNFHNNDDEDDDGGESTSPVPEYSRHQKPVPPRFQKQQ----------QQQEQVYKL      662
frog            -----LKAETSSEEEYAEPTSPAQDFSKHQKLIPPRFQRQQ----------QQQEQLYKM      655
chicken         --------QSSSEDELREAPSPAQEFSKYQKSLPPRFQRQQ-----------QQEQLYKM      638
human           NSS---------EEEAREAGSPAQEF-KYQKSLPPRFQRQQQQ--------QQQEQLYKM      643
mouse           NSSSSSSSSSSIEEEVRESGSPAQEFSKYQKSLPPRFQRQQQQQQQQQQQQQQQEQLYKM      660
                            :::  *  **. :: ::** :*****:**           ****:**:

zebrafish       ASWQQSAH--PAQPSSAHPQRGFYPPH--VLGFDPRWMMMPPYMDPRLAQGCSPVDYYPP      718
frog            QQWQQQQQQAYVASTHSNPPRTFYSPHPQMLGFDPRWMMMPSYMDPRMTQGRAPVDFYPS      715
chicken         QHWQQQQV----YPPPSHSHRTFYPPHPQMLGFDPRWMMMPSYLDPRMAQSRTPVDFYPS      694
human           QHWQ--PV----YPPPSHPQRTFYPHHPQMLGFDPRWMMMPSYMDPRITPTRTPVDFYPS      697
mouse           QHWQ--PV----YPPPSHPQRTFYPHHPQMLGFDPRWMMMPSYMDPRITPTRTPVDFYPS      714
                  **            ::  * **  *  :*********** *:***::   :***:**

zebrafish       GVHSSGLVKPVIQPDHLNSPGST--SDEGCHPSMHQERRAPSTEPYQVWNQDSYPSARSF      776
frog            SIHPPGVMKHMIQQDSMSGSGSCHSDEQNCQSE----RRPQSAEPMSGWGQESYVQLHSK      771
chicken         ALHPSGIMKPMIQQDSIGGN-SCRSEDQNCQAG-QVERKAAPMDPVPVWGQESYASLQSK      752
human           ALHPSGLMKPMMPQESLNGT-GCRSEDQNCVPP-LQERKVTPIDSPPVWSPEGYMALQSK      755
mouse           ALHPSGLMKPMMPQESLSGT-GCRSEDQNCVPS-LQERKVTALDPAPVWSPEGYMALQNK      772
                .:*  *::* ::  : :..  .   .::.*       *:    :    *. :.*   :.

zebrafish       TPPYQ--RTHENGER-----------APADDRSDRSTSRHDSYEEHRHERTDSPAEETTH      823
frog            ACSLPQQKQAETAAV-GSYSRNESSYSSAREKSDGSPTHPEPLEREEEYLS-VSYEKKST      829
chicken         GYSLSHQKQSDNMTMEGLHARNGSSYSSSPGRPENLNTQRDLFEERGEEYL-NAFDKKTQ      811
human           GYPLPHPKSSDTLAM-DMRVRNESSFSASLGRAGGVSAQRDLFEERGEEYL-SAFDKKAQ      813
mouse           GYSLPHPKSADTLAM-GMHVRNERSYCASPGRPGGISAQRDLFEERGEEFL-SAFDKKAQ      830
                       :  :.              . :  :     :: :  *.. .       ::.:
```

```
zebrafish       --------------------------QGFRQGR---VSDGSQREPMMLSRT---------      845
frog            GSFSSCISPQRRGQDALYQHLESGIESGVNRGRQMAARDSSSRGSEFIKTDKKPQFNGWG      889
chicken         GDFDSCLSSQRLGQDLLFQHQESVQETCPSSNRHANL-RCSPLEPDFIQAEKKPEYNGWD      870
human           ADFDSCISSQRIGQELLFPPQENVQDAGAPGGHTQNL-RCSPLEPDFVPDEKKPECGSWD      872
mouse           ADFDSCISSQRIGQELLFPPQENVQEVGAPGGCTPNL-RCSPLEPDFVPAEKKPEYGSWD      889
                               .        *     ::

zebrafish       ------HDDAQHQKEEPYDSKEKSYEADFWRRDVRKE----SSGQTQWSDHGSSSSSSIS      895
frog            YAPHQKSSETASRAEEDLSKEDLSDELEHWKKEDGGSSQDG-TGETCWRSESANSN--SQ      946
chicken         ISHHQKPSEPAAEITEEVPRNEQSFAADPWKKEGASNTKQPAEETAEWAPENR-ST--GV      927
human           VSHQPETADTAHGVERETPREGTAFNISSWDKNGSPNKQP--SSEPEWTPEPR-SS--SS      927
mouse           VGHQPKAADTANGVELEAPRQEPSFHVSSWEKEGSPKKQP--NPEPEWTPEPR-SS--SG      944
                        :           :  :   . * ::   .          *  .   *.

zebrafish       QPSETSGRTLRRTGPIKKPVLKPLKVEDKENEKPKAEPEEKPV--PYRLEKEV--ITNVY      951
frog            QQSEHLGR-SRRSGPIKKPVLKALKIEDKELEKNKA--EIKEPVKPFKE---KI-LPKTE      999
chicken         QQQEQIGR-TRRSGPIKKPVLKALKVEEKEKEMEKVKLEGEDTSRHL-KE--KVTVQKVE      983
human           QHPEQTGR-TRRSGPIKKPVLKALKVEDKEKELEKIKQELGEESTRLAKEKEQSPTAEKD      986
mouse           QHQEQPGR-TRRSGPIKKPVLKALKVEEKEKALERGRQGLREESSQRAPEKEP--VGRAE      1001
                *  *  **  **:********* **:*:**    :                      .

zebrafish       DLKKDAPHVSNRHSSSVEDKQADALKMEKT---------SALSEELHKESCWEKSQSDCSD      1003
frog            CVVKTEPVVSSPPPSMIEVKTPPPPSPVQE---------TDKFPQEKAERSWETKPLR--E      1049
chicken         NESDDSAALLNSTRYLLDDKGSSQASLAREAEKSQEEEEEEEEEDKPERTWENKVSR--E      1041
human           EDEENDASLANSSTTTLEDKGPGHATFGREATKF-------EEEEKPDKAWEARPPR--E      1037
mouse           EDEENNPALANA-SSALEDKAASRAGFAHEASKL-------DEDEKADKTWESRPSR--E      1051
                   .    : .     :: *        :              : : :  **       :

zebrafish       SRDPTAPRRNNWIFIDEEQAFAGA-RGRGRGRGFREFNSRGGGARGGRSDTNRG--AYNN      1060
frog            SSSTSLQKRSDWIFIDEEQAFGGRTQGRGRGRGFKDFGFRNRGVSG--------------      1095
chicken         SSELPPTKRNNWIFIDEEQAFGGRGQGRGRGRGFREFTFRGRG------------TVVSS      1089
human           SSDVPPMKRNNWIFIDEEQAFGVRGQARGRGRGFREFTFRG-RPAGGNGSGLCGGGVLGA      1096
mouse           ASDIPPTKRNNWIFIDEEQAFGGRGQARSRGRGFREFTFRGGRPAGSSTSGLCGTGVLGS      1111
                : .    :*.:**********.   :.*.*****::*  *.

zebrafish       NNNNTGAQRLTRGRGNRVFR-GDDLQRGKPRRRNVSETHSETSEYEEQPKRPRQKVSENG      1119
frog            --TY-SGQKVSRGRGVREYNPPEDLRGKASRRRIASETHSEGSEYEELPKRRRQRGTENG      1152
chicken         RGVYN-NQRSSRGRGLREFNQPEDFPRGKPRRRIASETHSEGSEYEELPKRRRQRGSENS      1148
human           RSIYCSSQRSGRGRGLREFARPEDCPRAKPRRRVASETHSEGSEYEELPKRRRQRGSENG      1156
mouse           RGMYSSGQRSNRGRGLRDFPPPEDCPRAKPRRRIASETHSEGSEYEELPKRRRQRGSEHS      1171
                       *:  **** * :   :*      *** .****** ***** *** **: :*:.

zebrafish       GEASGE-------VKKADRESWRSNKVYTDDQSEK----PKSRVFGRSFPPRLNA-AYNR      1167
frog            NDPAVQEREAEDLRKGDFQDSWRSNRNYSDDQNNTDSKSRAPRAFGRSLPPRLSN-SYGR      1211
chicken         NDGSVLDREDSDLKKGDFKESWRSNKIYSDDHTSLDPKMRAPRAFGRSLPPRLSNSGYGR      1208
human           NEGSLLEREESTLKKGDCRDSWRSNKGCSEDHSGLDAKSRGPRAFGRALPPRLSNCGYGR      1216
mouse           HEGMLTERDEG-----ALKDSWRSNRTYTEDQGGIDTRSRGSRTCGRALPPRLSNCSYGR      1226
                 :                ::*****:  ::*:          *. **::****.  .*.*

zebrafish       GFTGTRDISTWRGRGTQF-----GS-------SSSQENGYNFVADSYSKRTEPLKYPAKF      1215
frog            RPLSTKESSHWQAKSGGSSWQEYGA--PAEAYGSRHHADRDYAH-DY-------RYADSF      1261
chicken         RGFMGKEPTQWQGRSGGAGWQEYSHTSPSDGFGSRQQSDRDYIQDSY-------KHVDSF      1261
human           RTFVSKESPHWQSKSPGSSWQEYGP---SDTCGSRRPTDRDYVPDSY-------RHPDAF      1266
mouse           RTFVAKEPPHWQSRSPGSSWQEYGS---SDPCGPRRGTDRDYIPDSY-------RQSDTF      1276
```

```
                     ::   *:.:.        .        .  :  . ::   .*       :    *

zebrafish      TGTFAENGVEDRDGGYY------VDDNPDNRPLRRRRPPRQDKPPRFRRLRQERDGAGAW    1269
frog           PSRGFDESHGDERRSFFQEEYS-DRDSLEKRSFGRRRPPRQDKPPRFRRLRQERDPLGQW    1320
chicken        SSRVFDESHLDDKRHFFQEDYLADQENIENRPFRRRRPPRQDKPPRFRRLRQERELVGQW    1321
human          GGRGFEDSRAEDKRSFFQDEHVADSENAENRPFRRRRPPRQDKPPRFRRLRQERESLGLW    1326
mouse          GSRLFEDSRTEDKRSFFQDDHGADSENAENRPFRRRRPPRQDKPPRFRRLRQERESLGLW    1336
                .   ::.  :    ::        :. ::* : ********************:  * *

zebrafish      SNDDYV-NGEFPNQWPSRAKSAEEHWQNHYPGGRS---QSQEMTAQSQGEDWETGSDNSD    1325
frog           SGEETVGTGNSSEHWQSRPRVPLNDKSG--PVHRRSPEMSHHN-SDHVTEDWETASESSD    1377
chicken        NPEEG-GPNLLPSQWPGRPKLSTTEKSS--ISGRRSPELSYQNSSDHANEEWETASESSD    1378
human          GPEEE--PHLLAGQWPGRPKLCSGDKSG--TVGRRSPELSYQNSSDHANEEWETASESSD    1382
mouse          GPEEE--SHLLASQWPGRSKLCPGDKSG--PGGHRSPELSYQNSSDHANEEWETASESSD    1392
               . ::         :* .* :    . ..     :     * .  ::   *:***.*:.**

zebrafish      FGDWREKRGPHGD-PL---TDAALGDPGSEKRELSKRSFSSQRPLD-RQNRKNDVSVMEN    1380
frog           FSEKRPG--------DMDGDGSQSGSGLSEKRELSKRSFSSQRPLMDRQSRKVEPSGYDE    1429
chicken        FSERRERRDGVS-ESEGQLESGLGSGSLGEKRELAKRSFSSQRPLVDRQNRKAEPAGYAE    1437
human          FSERRERREGPGSEPDSQVDGGLSGASLGEKKELAKRSFSSQRPVVDRQSRKLEPGGFGE    1442
mouse          FSERRERREGLVAEPEAQGDGGLSGSSLGEKKELAKRSFSSQRPLADRQSRKLEPGGFGE    1452
               *.: *               ..  .   .**:**:*********:  **.** : .   :

zebrafish      SK-MGRPADAGSRNDSWQNGVASGSKRSPEESVSGL-------SSASVYGVEQNEDRS--    1430
frog           KPSRVSGSS--SRNDFQKSSGPLKSSRCSDDSYG-------AESSHHRYAMDRSMHCDSG    1480
chicken        QSVRTVGA--ASRYESQQNGTLIKSKRSPEE--GGGLGNTSGGSSHSIYSLDRASLANSE    1493
human          KPVRPGGGDTSPRYESQQNGTPLKVKRSPDEALPGGLSGCSSGSGHSPYALERAAHASAD    1502
mouse          KPVRPGGGEPSPRCESQQSGTPLKVKRSPDEALPGGLG------SHSPYALERTTHASSD    1506
               .      .    * :  :..     .*. ::             .   *.:::    .

zebrafish      TPEPTGKIIEKELKPRN-MKADMTEPLSQYDLNTYSIESDSG---------ASVPSPEVF    1480
frog           E---AGKKQERDSRTPGLKSSDKNEVIGQFDLKYGDSMLEEE-------SEV--------    1522
chicken        SAEGPGKKSEKEPKSAVQRASEKGETLSQFELNYGSTIIDNRVSSTAEENEVGSMAGEGF    1553
human          LPEASSKKAEKEAKLAAPRAGEQGEAMKQFDLNYGSAIIENCGSSPGEESEVGSMVGEGF    1562
mouse          GPETPSKKSEREVSLPTQRASEQEEARKQFDLGYGNALIDNCASSPGEENEASSVVGEGF    1566
                    .*  *::        .:  *   *::*   .   :.          .

zebrafish      QDSLSKKQRRPQEDERRRKEQGPPSSVKSRTIASKMPPRFAKKQGSMSMEQPEETLTANN    1540
frog           -GDIGNKPRRVLEKDRRKKDQVVQVPAKGSSIQSRIPPRFAKKQNGMCLDQADVTA----    1577
chicken        IEVLTKKQRRLLEEERRKKEQAAQAPAKARVLQSRIPPRFAKKQNSLCLEQSDVTVSGNS    1613
human          IEVLTKKQRRLLEEERRKKEQAVQVPVKGRGLSSRIPPRFAKKQNNLCLEQGDVTVPGSS    1622
mouse          IEVLTKKQRRLLEEERRKKEQAAQVPVKGRGLSSRIPPRFAKKQNGLCLEQ-DVTVPGSS    1625
                  : :* **  *.:**:*:*     .*.  : *::********..:.::* : *

zebrafish      LGTEIWETNSTALTVQSSGGDSWTKQVSYTGSEP--------------------------    1574
frog           --KEIWESSGQGISVQ-PGTDTWSKPVSSFSTES--SSTEGFKGSQGDSGIDLSAESRES    1632
chicken        LGTEIWESNSPALSVQSPGSDSWSKPVNTFNGTESSSTEQGFKGSQGDSGIDLSAESRES    1673
human          LGTEIWESSSQALPVQAPANDSWRKAVTAFSSTETGSAEQGFKSSQGDSGVDLSAESRES    1682
mouse          LGTEIWENSSQALPVQGAASDSWRTAVTAFSSTEPGT-SEGFKSSQGDSGVDLSAESRES    1684
                 .****... .: **  . *:* . *.  .

zebrafish      ------------------------------------------NSEDSDAGPEQSKEHKPGPI    1594
frog           SATSSQRSSPYGTMKPEEMNGTGVAEPKPDCPKEQGQKQSEKKDSDPGSGLNKEHKPGPI    1692
chicken        SATSSQRSSPYGTLKPEEMNGAGLVDPKPDCQKEQVQKQSDKKDSDQGSGQNKEHKPGPI    1733
human          SATSSQRSSPYGTLKPEEMSGPGLAEPKADSHKEQAPKPSEQKDSEQGSGQSKEHRPGPI    1742
```

```
mouse           SATSSQRSSPYGTLKPEEISGPGLAESKADSHKDQAQKQAEHKDSEQGSAQSKEHRPGPI      1744
                                                        : :**: *   .***:****

zebrafish       GNERSLKNRKGSEGLERLEGS-ITPVNGVDIHVENVIPVPPIEFGVNAKDSDFSLPAGSA      1653
frog            GNERSLKNRKGSEGTERLEGG-VPPVNGVEIHVDSVLPVPPIEFGVSAKDADYALPPGTA      1751
chicken         GNERSLKNRKGSEGTERLEGN-IPPVNGVEIHVDSVLPVPPIEFGVNPKDSDFSLPPGSA      1792
human           GNERSLKNRKGSEGAERLQGAVVPPVNGVEIHVDSVLPVPPIEFGVSPKDSDFSLPPGSA      1802
mouse           GNERSLKNRKGSEGAERLPGAVVPPVNGVEIHVDSVLPVPPIEFGVSPKDSDFSLPPGSV      1804
                ************** *** *  : *****:***:.*:*********. **:*::** *:.

zebrafish       AVPVSNPVSKLQDALAGNAGLTQAIPMLRRDH-LQPAITLNPISFPSADLTLKMESARKA      1712
frog            TVQAANTVTKLQDALASKAGLTQSIPILRRDHHMQQGMSLN-MSYPTADLTLKMESARKA      1810
chicken         SGTAANPVTKLQDALASNAGLTQSIPILRRDHHLQRCIGLNPMSFPTAELTLKMESARKA      1852
human           SGPTGSPVVKLQDALASNAGLTQSIPILRRDHHIQRAIGLSPMSFPTADLTLKMESARKA      1862
mouse           SGPVGNPVAKLQDVLASNAGLTQSIPILRRDHHMQRAIGLSPMSFPTADLTLKMESARKA      1864
                :  ... * ****.**.:*****:**:***** :*  : *. :*:*:*:***********

zebrafish       WENSQAVPEQGSPGS-GSSAQPVCSVGSS-SVS---YSSFSMPPMPVASVAPSVSMQGSH      1767
frog            WENSPSLPEQNSPAGPGSGIQPPSSVGASTGVNYSSFGGVSMPPMPVASVAPSASMPGNH      1870
chicken         WENSPSLPEQNSPGGAGSGIQPPSSVGASNGVSYSSFGGVSMPPMPVASVAPSASIPGNH      1912
human           WENSPSLPEQSSPGGAGSGIQPPSSVGASSGVNYSSFGGVSMPPMPVASVAPSASMPGSH      1922
mouse           WENSPSLPEQSSPGGAGSGIQPPSSVGASNGVNYSSFGGVSMPPMPVASVAPSASIPGSH      1924
                **** ::***.**.. **. ** .***:* .*.   :...*************.*: *.*

zebrafish       IPPLYLDGHVFPSQPRLVPPL-TQQPSYQQ-ATPQQIPISLHTSLQAQAQLGLRGALPVS      1825
frog            IPPLYLDGHVFTNQPRLVQQTIPQQQGYQQAAAAQQIPISLHTSLQAQAQLGLRGGLPVS      1930
chicken         IPPLYLDGHVFASQPRLVPQTIPQQQSYQQAAAAQQIPISLHTSLQAQAQLGLRGGLPVS      1972
human           LPPLYLDGHVFASQPRLVPQTIPQQQSYQQAAAAQQIPISLHTSLQAQAQLGLRGGLPVS      1982
mouse           LPPLYLDGHVFASQPRLVPQTIPQQQSYQQAATAQQIPISLHTSLQAQAQLGLRGGLPVS      1984
                :********** .*****     ** .*** *: *********************.****

zebrafish       QSQEIFSSIPPFRSQVYMHPNLSQASPMVLSGGAALKGPYSAFPGLQPSDLVKSQSGSHY      1885
frog            QSQEIYSSMQPFRSQVYMHPSLSQPSAMVLTSGTGLKPPYNPFPGMQTLEMVKTQPTSPY      1990
chicken         QSQEMYSSIQPFRSQVYMHPSLSQPSTMVLTGGTALKPPYSAFPGMQPLEVVKTQSGSPY      2032
human           QSQEIFSSLQPFRSQVYMHPSLSPPSTMILSGGTALKPPYSAFPGMQPLEMVKPQSGSPY      2042
mouse           QSQEIFSSLQPFRSQVYMHPSLSPPSTMILSGGTALKPPYSAFPGIQPLEMVKPQSGSPY      2044
                ****::**: **********.**  * *:*:.*:.** **. ***:*  ::** *  * *

zebrafish       QPINGSTPLVYD----QPTGMSTSQLMDSQLIQVTMPMPGSQL---RYGSAQQHLILPQS      1938
frog            QPLNGSQQLVYESQLNQA-----SQMMDSQLTQLTMPMPGSQLQMPRYSSGQQTMILPQS      2045
chicken         QPLNGSQTLVYEGQINQAAGMGASQMMDSQLTQLTMPVPGSQLPLPRYGSGQQPLILPQS      2092
human           QPMSGNQALVYEGQLSQAAGLGASQMLDSQLPQLTM-------PLPRYGSGQQPLILPQS      2095
mouse           QPMSGNQALVYEGQLGQAAGLGTSQMLDSQLPQLTM-------PLPRYGSGQQPLILPQS      2097
                **:.*.  ***:    *      **::**** *:**          **.*.** :*****

zebrafish       IQLQQNQNLSVGAPRRMMPPGSQP-VLSGSREVSQMDMKGFQFSDKPSHSPGI----PSG      1993
frog            IQLPQGQNLPVGAPRRMQP------SMLTSRESSQMEMKSFHFTDGKQNMPTAMQ--AQH      2097
chicken         IQLPQGQNLSVGAPRRILPPGSQPSVLAASRESSQMEMKGFHFSDGKQNMSSGGSVPSPH      2152
human           IQLPPGQSLSVGAPRRIPPPGSQPPVLNTSREPSQMEMKGFHFADSKQNVPSGGPVPSPQ      2155
mouse           IQLPPGQSLSVGAPRRVPPPGSQPPVLNTSRESAPMELKGFHFADSKQNVPTGGSAPSPQ      2157
                ***  .*.* ******: *       :  *** : *::*.*:*:*  .:

zebrafish       SYR-------------PGSASPSGKASGAP---AVASLAGHYTQQVSGPQGSMVMHMRPP      2037
frog            SYR-------------PSSASPSGKSPGPGPSANLGSVQGHYPQQVKHRSDDSKGGLR--      2142
chicken         AYRIYSMNVVDSSISRPSSASPNGKPS--GPAVSMGSVQGHYVQQAKQRVDENKANLG--      2208
```

```
human           TYR-------------PSSASPSGKPS--GSAVNMGSVQGHYVQQAKQRVDEK-PSLG--     2197
mouse           AYR-------------PSSASPSGKPS--GSAVNMGSVQGHYVQQAK-RVDEK-PGLG--     2198
                :**             *.****.**       :.*: *** **..   ..    :

zebrafish       SSGPFPTPIQRPIMQVNKPVIIRSPPYPGPNTGPAHTHAHTHNPEGPGKGPEDGLKECVA     2097
frog            ------------------------------------------------------SPKVPE     2148
chicken         ------------------------------------------------------AVKLQE     2214
human           ------------------------------------------------------AVKLQE     2203
mouse           ------------------------------------------------------TVKLQE     2204
                                                                      :

zebrafish       SAAPVKASASRSGAIKPQPLKLEEGKA     2124
frog            QPSASPMKPARTGAIKPQAVKVEESKA     2175
chicken         TASTSQMKPVRTGAIKPQAVKVEESKA     2241
human           APSAA-SQMKRTGAIKPRAVKVEESKA     2229
mouse           ASSAT-SQMKRTGAIKPRAVKVEGSKA     2230
                   :   .  *:*****: :*:* .**
```

Extended Data 2. Sequence alignment of PRRC2A, PRRC2B, and PRRC2C proteins in humans was shown.

```
PRRC2A[Homo_sapiens(human)]        MSDRSGPTAKGKDGK-KYSSLNLFDTYKGKSLEIQKPAVAPRHGLQSLGKVAIARRMPPP    59
PRRC2B[Homo_sapiens(human)]        MSDRLGQITKGKDGKSKYSTLSLFDKYKGKSVDAIRSSVIPRHGLQSLGKVAAARRMPPP    60
PRRC2C[Homo_sapiens(human)]        MSEKSGQSTKAKDGK-KYATLSLFNTYKGKSLETQKT--TARHGLQSLGKVGISRRMPPP    57
                                   **:: *  :*.**** **::*.**:.*****::  :     **********. :******

PRRC2A[Homo_sapiens(human)]        ANLPSLKAENKGNDPNVSLVPKDGTGWASKQEQSDPKSSDASTAQPPESQPLPASQTPAS    119
PRRC2B[Homo_sapiens(human)]        ANLPSLKSENKGNDPNIVIVPKDGTGWANKQDQQDPKSSSATASQPPESLPQPGLQKSVS    120
PRRC2C[Homo_sapiens(human)]        ANLPSLKAENKGNDPNVNIVPKDGTGWASKQEQHEEEKTP----EVPPAQPKPGVA----    109
                                   *******:********: :*********.**:* : :.:     : * : * *.

PRRC2A[Homo_sapiens(human)]        NQPKR-PPAAPENTPLVPSGVKSWAQASVTHGAHGDGGRASSLLSRFSREEFPTLQAAGD    178
PRRC2B[Homo_sapiens(human)]        NLQKPTQSISQENTNSVPGGPKSWAQLNGKPVGHEGGLRGSSRLLSFSPEEFPTLKAAGG    180
PRRC2C[Homo_sapiens(human)]        ------------APPEVAPAPKSWASN--KQGGQGDGIQVN----SQFQQEFPSLQAAGD    151
                                                   *  . ****.   .  .: .* : .        :***:*:***.

PRRC2A[Homo_sapiens(human)]        QDKAAKERESAEQSSGPGPSLRPQNSTTWRDGGGRGPDELEGP----------DSKLHHG    228
PRRC2B[Homo_sapiens(human)]        QDKAGKEKGVLDLSYGPGPSLRPQNVTSWREGGGRHIISATSLSTSPTELGSRNSSTGDG    240
PRRC2C[Homo_sapiens(human)]        QEKK--EKETNDDNYGPGPSLRPPNVACWRDGGKAAGSPSSSDQ-DEKLPGQDESTAGTS    208
                                   *:*   *:   : . ******** * : **:**        .           :*.   .

PRRC2A[Homo_sapiens(human)]        HD--------PRGGLQPS-----------G-PPQFPPYRGMMPPFMYPPYLPFPPPYGPQ    268
PRRC2B[Homo_sapiens(human)]        APSSACTSDSKDPSLRPAQPVRKGASQFMGNVYHPPTYHDMLPAFMCSPKSSENQGTVER    300
PRRC2C[Homo_sapiens(human)]        EQNDILKVVEKRIACGPPQAKLNGQ-----QAALASQYRAMMPPYMFQQYPRMTYPP-LH    262
                                                .  *                    *: *:* :*             :

PRRC2A[Homo_sapiens(human)]        GPYRYPT--------------PDGPSRFPRVAGPRGSGPPMRLVEPVGRPSILKEDNLKE    314
PRRC2B[Homo_sapiens(human)]        GSFPLPQLRLEPRVPFRQFQMNDQDGKENRLGLSRPLRPLRQLVERAPRPTIINAENLKG    360
PRRC2C[Homo_sapiens(human)]        GPMRFPPS------------------LSETNKGLRGRGPPPSWASEPERPSILSASELKE    304
                                   *    *                            *   *    ..   **:*:. .:**

PRRC2A[Homo_sapiens(human)]        ---FDQLDQENDDGWAGAHEEVDYTEKLKFSDEEDGRDSDEEGAEGHRDSQSASGEER-P    370
PRRC2B[Homo_sapiens(human)]        ---LDDLDADADDGWAGLHEEVDYSEKLKFSDDEEEEEVVKDGRPKWNSWDPRRQRQLSM    417
PRRC2C[Homo_sapiens(human)]        LDKFDNLDAEADEGWAGAQMEVDYTEQLNFSDDDEQGSNSPKENNSEDQGSKASENNENK    364
                                      :*:** : *:**** : ****:*:*:***:::  .   .      . .    .:

PRRC2A[Homo_sapiens(human)]        PEADGKKGN----------SPNSEPPTPKTAWAETSRPPETEPGPPAPKPPLPPPHRGPA    420
PRRC2B[Homo_sapiens(human)]        SSADS--------------ADAKRTREEGKDWAEAVGASRVVRKAPDPQP-----PPRKL    458
PRRC2C[Homo_sapiens(human)]        KETDEVSNTKSSSQIPAQPSVAKVPYGKGPSFNQERGTSSH----------LPP-PPKLL    413
                                    .:*               :  .        : :

PRRC2A[Homo_sapiens(human)]        GNWGPPGDYPDR--GG-PPCKPPAPEDEDEAWRQRRKQSSSEISLAVERARRRREEEERR    477
PRRC2B[Homo_sapiens(human)]        HGWAPGPDYQKS--SMGSMFRQQSIEDKEDKPPPRQKFIQSEMSEAVERARKRREEEERR    516
PRRC2C[Homo_sapiens(human)]        AQQHPPPDRQAVPGRPGPFPSKQQVADEDEIWKQRRR-QQSEISAAVERARKRREEEERR    472
                                       *  *                  *:::    *::  .**:* ******:********

PRRC2A[Homo_sapiens(human)]        MQEERRAACAEKLKRLDEKFGAPDKRLKAEPAAPPAAPSTPAPPPAVPKELPAPPAPP--    535
PRRC2B[Homo_sapiens(human)]        AREERLAACAAKLKQLDQKCKQARKAGEARKQAEKEVPWSPSAEKASPQEN----G----    568
PRRC2C[Homo_sapiens(human)]        MEEQRKAACAEKLKRLDEKLGILEKQPSPEEIRERERKEREREKELEKEQEQEREKERE    532
                                    .*:* **** ***:**:*     *  . .                  :*
```

```
PRRC2A[Homo_sapiens(human)]      --PA---------------SAPTP---ETEPEE----------PAQAPPAQS--------    557
PRRC2B[Homo_sapiens(human)]      --PA------------------VH---KGSP-----------------------------    576
PRRC2C[Homo_sapiens(human)]      KDRERQQEKEKELEKEQEKQREMEKERKQEKEKELERQKEKEKELQKMKEQEKECELEKE    592
                                                            : .

PRRC2A[Homo_sapiens(human)]      ------TPTPGVAAAPTLVSGGGSTSSTSSGSFEASPVEPQLPSKEGPEPPEEVPPPTTP    611
PRRC2B[Homo_sapiens(human)]      ----------------------------------------EFPAQETPTTFPEEAPTVSP    596
PRRC2C[Homo_sapiens(human)]      REKLEEKIEPREPNLEPMVEKQES---ENSC---NKEEEPVFTRQDSNRSEKE----ATP    642
                                                                          :  ::      *    .:*

PRRC2A[Homo_sapiens(human)]      PVPKVEPKGDGIGPTRQPPSQGLGYPKYQKSLPPRFQRQQQEQLLKQQQ-QHQWQQHQQG    670
PRRC2B[Homo_sapiens(human)]      AVAQSNSSEE----EAREAGSPAQEFKYQKSLPPRFQRQQQQQQQEQLYKMQHWQPVY--    650
PRRC2C[Homo_sapiens(human)]      VVH---ETEPESGSQPRPAVLSGYFKQFQKSLPPRFQRQQEQM------KQQQWQQQQQQ    693
                                  *     .        :         ::************::         ::**

PRRC2A[Homo_sapiens(human)]      SAPPTPVPPSPPQPVTLGAVPAPQAPPPPPKALYPGALGRPPPMPPMNFDPRWMMIPPYV    730
PRRC2B[Homo_sapiens(human)]      --P----PP-----------------SHPQRTFYPH------HPQMLGFDPRWMMMPSYM    681
PRRC2C[Homo_sapiens(human)]      GVLPQTVPSQPS---------SSTVPPPPHRPLYQPMQPHPQHLASMGFDPRWLMMQSYM    744
                                        *                    * : :*            :.*****:*:  *:

PRRC2A[Homo_sapiens(human)]      DPRLLQGRPPLDFYPPG---VHPSGLVPRERSD--SGGSS--SEPFDRHAPAMLRERGTP    783
PRRC2B[Homo_sapiens(human)]      DPRITPTRTPVDFYPSA---LHPSGLMKPMMPQESLNGTG--CRSEDQNCVPPLQERKVT    736
PRRC2C[Homo_sapiens(human)]      DPRMMSGRPAMDIPPIHPGMIPPKPLMRRDQ----MEGSPNSSESF-EHIARSARDHAIS    799
                                 ***:   *  :*: *     : *. *:          *:   ..   .:     :::

PRRC2A[Homo_sapiens(human)]      PVDPKLAWVGDVFTATPAEPRPLTSPLRQAADEDDKGMRSETPP----------------    827
PRRC2B[Homo_sapiens(human)]      PIDSPPVWSPEGYMALQSKGYPLPHPKSSDTLAMDMRVRNESSFSASLGRAGGVS-----    791
PRRC2C[Homo_sapiens(human)]      LSEPRMLWGSDPYPHAEPQQA--TTPKAT---EEPEDVRSEAALDQEQITAAYSVEHNQL    854
                                   :    *  : :     :      *           :*.*:

PRRC2A[Homo_sapiens(human)]      ---------VPPPPPYLASYP------------------------------------GFP    842
PRRC2B[Homo_sapiens(human)]      ---AQRDLFEERGEEYLSAFDKKAQAD----------FDSCISSQRIGQELLFPPQENVQ    838
PRRC2C[Homo_sapiens(human)]      EAHPKADFIRESSEAQVQKFLSRSVEDVRPHHTDANNQSACFEAP--DQKTLSAPQEER-    911
                                                 :  :

PRRC2A[Homo_sapiens(human)]      ENGAPGPPISR---------FPLEEPGPRPLPWPPGSDEVAKIQTPPPKKEPPKEETAQ-    892
PRRC2B[Homo_sapiens(human)]      DAGAPGGHTQNLRCSPLEPDFVPDEKKPECGSWDVSHQPETADTAHGVERETPREGTA--    896
PRRC2C[Homo_sapiens(human)]      --------ISAVESQPSR---------KRSVSHGSNHT-----QKPDEQRSEPSAGIPKV    949
                                          .                  .      .            ::. *

PRRC2A[Homo_sapiens(human)]      ----------LTGPEAG-RKPARGVGSGGQGPPPPRRESRTETRWGPRPGSSRRGIPPEE    941
PRRC2B[Homo_sapiens(human)]      ----------------------FNISS-WDKNGSPNKQPSSEPEWTPEPRSSSSQHPE--    931
PRRC2C[Homo_sapiens(human)]      TSRCIDSKEPIERPEEKPKKEGFIRSSEG-PKPEKVYKSKSETRWGPRPSSNRREEV---    1005
                                                          .*          :  :* .* *.* *.

PRRC2A[Homo_sapiens(human)]      PGAPPRRAGPIKKPPPPTKVEELPPKP-----------------LEQG---DETPKPPKP    981
PRRC2B[Homo_sapiens(human)]      QTGRTRRSGPIKKPVLKALKVEDKEKELE---K-------IK--QELG---EESTRLAKE    976
PRRC2C[Homo_sapiens(human)]      NDRPVRRSGPIKKPVLRDMKEEREQRKEKEGEKAEKVTEKVVVKPEKTEKKDLPPPPPPP    1065
                                      **:******       *   :                   *     :

PRRC2A[Homo_sapiens(human)]      D-----------------------------------------------------PLKITK    988
PRRC2B[Homo_sapiens(human)]      KEQSP------------TAEKDEDE-----------ENDASLANSSTTTLEDKGPGHATF    1013
PRRC2C[Homo_sapiens(human)]      QPPAPIQPQSVPPPIQPEAEKFPSTETATLAQKPSQDTEKPLEPVSTVQVE---PAVKTV    1122
                                 .                                                     *   *
```

```
PRRC2A[Homo_sapiens(human)]      --GKL-GG----------P-K----ETPPNGNLSP------APRLRRDYS-------YER      1017
PRRC2B[Homo_sapiens(human)]      --GRE-ATKFEEEEKPDKAWE----ARPPRESSDV------PPMKRNNWIFIDEEQAFGV      1060
PRRC2C[Homo_sapiens(human)]      NQQTMAAPVVKEEKQPEKVISKDLVIERPRPDSRPAVKKESTLPPRTYWKEARERD---W      1179
                                       .             .       *. .             *  :

PRRC2A[Homo_sapiens(human)]      VGPTSCRGRGRGEYFARGRGFRGT----YGGRGRGARSREFRSYREFRGDDGRGGGTG--      1071
PRRC2B[Homo_sapiens(human)]      RGQARGRGRGFREFTFRGRPAGGNGSGLCGGGVLGARSIYCSSQ-----RSGRGRGLREF      1115
PRRC2C[Homo_sapiens(human)]      FPDQGYRGRGRGEYYSRGRSYRGS----YGGRGRGGRGHT-RDYPQYRDNKPR-------      1227
                                       ****  *:  ***   *.     **   *.*.    .       . *

PRRC2A[Homo_sapiens(human)]      ---GPNHPPAPRGRTASETRSEGSEYEEIPKRRRQRGSETGSETHESDLAPSDKEAPTPK      1128
PRRC2B[Homo_sapiens(human)]      ARPEDCPRAKPRRRVASETHSEGSEYEELPKRRRQRGSENGNEGSLLEREEST-----LK      1170
PRRC2C[Homo_sapiens(human)]      --AEHIPSGPLRQREESETRSESSDFEVVPKRRRQRGSETDTDSEIHESASD---KDSLS      1282
                                            * *  ***:**.*::* :**********...:    :   .       .

PRRC2A[Homo_sapiens(human)]      EGTLTQVPLAPPPPGAPP--------SPAPA----------RFTARGGRVFTPRGVPSRR      1170
PRRC2B[Homo_sapiens(human)]      KGDCRDSWR--SNKGCSE--------DHSGL----------DAKSRGPRAFGRALPPRLS      1210
PRRC2C[Homo_sapiens(human)]      KGKLPKREERPENKKPVKPHSSFKPDNHVRIDNRLLEKPYVRDDDKAKPGFLPKGEPTRR      1342
                                 :*   .                    .                  :.   *     *

PRRC2A[Homo_sapiens(human)]      --G----RGGGRP---P---------PQVCPGWSPPAKSLAPKKPPTGPLPPSKEPLKEK      1212
PRRC2B[Homo_sapiens(human)]      NCG----YGRRTF---V---------SKESPHWQSKSPGSSWQEYGPSDTCGSRRPTDRD      1254
PRRC2C[Homo_sapiens(human)]      GRGGTFRRGGRDPGGRPSRPSTLRRPAYRDNQWNPRQSEVPKPE------DGEPPRRHEQ      1396
                                   *     *                       *.         :        .    ...

PRRC2A[Homo_sapiens(human)]      LIPGPLSP-VARGGSN-------------GGSNVGMEDGERPRRRRHGRAQQQDKPPRFR      1258
PRRC2B[Homo_sapiens(human)]      YVPDSYRHPDAFGGRGFEDSRAEDKRSFFQDEHVADSENAENRPFRRRRPPRQDKPPRFR      1314
PRRC2C[Homo_sapiens(human)]      FIPIA----------------ADKRPPKF--ERKFDPARERPRRQRPTRPPRQDKPPRFR      1438
                                  :*                            ..       . *  *  *  :********

PRRC2A[Homo_sapiens(human)]      RLKQERENAARG-SEGKPSLTLPASAPGPEEALTTVTVAPAPRRAAAKSPDLSNQ-NSDQ      1316
PRRC2B[Homo_sapiens(human)]      RLRQERESLGLWGPEEEPHL-LAGQWPGRPKLC----SGDKSGTVGRRSPELSYQNSSDH      1369
PRRC2C[Homo_sapiens(human)]      RLREREAASKSNEV-------VAVPTNGTVNNVAQEPVNTLGDISGNKTPDLSNQNSSDQ      1491
                                 **::..               :     *  :              . ::*:** * .**:

PRRC2A[Homo_sapiens(human)]      ANEEWETASESSDFTSERRGDKEAPPPVLLTPKAVGTPGGGGGGAVPGISAMSRGDLSQR      1376
PRRC2B[Homo_sapiens(human)]      ANEEWETASESSDFSERRERRE-GPGSE---------PDSQVDG---GL----SGASLGE      1412
PRRC2C[Homo_sapiens(human)]      ANEEWETASESSDFNERRERDEKKNADLNA-Q----------------TVVKVGENVLPP      1534
                                 **************...*.  :

PRRC2A[Homo_sapiens(human)]      AKDLSKRSFSSQRPGMERQNRRPGPGGKA-GSSGSS----------SGGGGG-----GPG      1420
PRRC2B[Homo_sapiens(human)]      KKELAKRSFSSQRPVVDRQSRKLEPGGFG-EKPVR-----------PGGGDT-----SPR      1455
PRRC2C[Homo_sapiens(human)]      KREIAKRSFSSQRPVD-RQNRRGNNGPPKSGRNFSGPRNERRSGPPSKSGKRGPFDDQPA      1593
                                  ::::*********   **.*:   *                      .*        *

PRRC2A[Homo_sapiens(human)]      GRTGP----------------GRGDKRSWPSPKNRSRPPEERPPGLPLPPPPPSSSAVFR      1464
PRRC2B[Homo_sapiens(human)]      Y----------------------ESQQNGTPLKVKRSPDEALPGGLSGCSSGSGHSPYA      1492
PRRC2C[Homo_sapiens(human)]      GTTGVDLINGSSAHHQEGVPNGTGQKNSKDSTGKK---REDPKPGPKKPKEKVDALSQFD      1650
                                                         :...  :  :     ::  **        .. : :

PRRC2A[Homo_sapiens(human)]      LDQVIH-------------------SNPAGIQQALAQLSSRQ------------------      1487
PRRC2B[Homo_sapiens(human)]      LERAAH-------------------ASA-DLPEASSKKAEKE------------------      1514
PRRC2C[Homo_sapiens(human)]      LNNYASVVIIDDHPEVTVIEDPQSNLNDDGFTEVVSKKQQKRLQDEERRKKEEQVIQVWN      1710
                                 *:.                       .  .: :. ::  .:.
```

```
PRRC2A[Homo_sapiens(human)]   -------G----SVTAPGGHPRHKPGLPQAP------------Q-------------GPS   1511
PRRC2B[Homo_sapiens(human)]   -------A----KLAAPR-------------------------A-------------GEQ   1525
PRRC2C[Homo_sapiens(human)]   KKNANEKGRSQTSKLPPRFAKKQATGIQQAQSSASVPPLASAPLPPSTSASVPASTSAPL   1770
                                     .    .   *                                        .

PRRC2A[Homo_sapiens(human)]   PR-------------PPTRY----------------EPQRVNSGLSS-------------   1529
PRRC2B[Homo_sapiens(human)]   GE-------------AMKQF----------------D---LNY-----------------   1536
PRRC2C[Homo_sapiens(human)]   PATLTPVPASTSAPVPASTLAPVLASTSAPVPASPLAPVSASASVSASVPASTSAAAITS   1830
                                               .                       .

PRRC2A[Homo_sapiens(human)]   --DP-HFEEPGPMVRGVGGTPRDSAGVS--------PFPPKR------------------   1560
PRRC2B[Homo_sapiens(human)]   ---------GSAIIENCGSSPGEESEVGSMVGEGFIEVLTKK------------------   1569
PRRC2C[Homo_sapiens(human)]   SSAPASAPAPTPILASVS-TPA---SV-TILASASIPILASALASTSAPTPAPAASSPAA   1885
                                          :: . . :*     *          .  .

PRRC2A[Homo_sapiens(human)]   -----------------------RERPP----------------RKPELLQEESLP-PPH   1580
PRRC2B[Homo_sapiens(human)]   -----------------------QRRLL----------------EEERRKKEQAVQVPVK   1590
PRRC2C[Homo_sapiens(human)]   PVITAPTIPASAPTASVPLAPASASAPAPAPTPVSAPNPAPPAPAQTQAQTHKPVQNPLQ   1945
                                                                           : .   .: :  * :

PRRC2A[Homo_sapiens(human)]   SSGFLGSKPE-------GPGPQAE-------S--RDTGTEALTPHIWNRLHTA-------   1617
PRRC2B[Homo_sapiens(human)]   GRGLSSRIPPRFAKKQNNLCLEQG-------D--VTVPGSSLGTEIWESSSQA-------   1634
PRRC2C[Homo_sapiens(human)]   TTSQSSKQPPPSIRLPSAQTPNGTDYVASGKSIQTPQSHGTLTAELWDNKVAPPAVLNDI   2005
                                .  .  *            :         .        :*  .:*:

PRRC2A[Homo_sapiens(human)]   ----TSRKSYRPSSMEPWMEPLSPFED------VAGTEMSQSDSGVDLSGDSQVSSGPCS   1667
PRRC2B[Homo_sapiens(human)]   ----LP---VQAPANDSWRKAVTAFSSTETGSAEQGFKSSQGDSGVDLSAESRESSATSS   1687
PRRC2C[Homo_sapiens(human)]   SKKLGPISPPQPPSVSAWNKPLTSFGSAPSSEGAKN----GQESGLEIGTDTIQFGAPAS   2061
                                        :  : . * : :: * .        .      :**:::. ::   .. .*

PRRC2A[Homo_sapiens(human)]   QRSSPDGGLKGAAEGPPKRPGGSSPLNAVPCEGPPGSEPPRRPPPAPHDGDRKELPREQP   1727
PRRC2B[Homo_sapiens(human)]   QRSSPYGTLKPEEMSGPGL---AEPKAD--------SHKEQAPKPSEQKDSEQGSGQSKE   1736
PRRC2C[Homo_sapiens(human)]   NGNENEVVPVLSEKSADKIPEPKEQRQ----------KQPRAGPIKAQKLPDLSPVENKE   2111
                              : ..          .        .             .  :      :.       ..:

PRRC2A[Homo_sapiens(human)]   LPPGPIGTERSQRTDRGTEPGP---------------------------------IRPSH   1754
PRRC2B[Homo_sapiens(human)]   HRPGPIGNERSLKNRKGSEGAERLQG-------AVVP-----------PVNGVEIHVDSV   1778
PRRC2C[Homo_sapiens(human)]   HKPGPIGKERSLKNRKVKDAQQVEPEGQEKPSPATVRSTDPVTTKETKAVSEMSTEIGTM   2171
                                *****.*** :. : .:                                       :

PRRC2A[Homo_sapiens(human)]   RPGPPVQFGTSDKDSDLRLVVG--DSLKAEKEL----------TASVTEAIPVSRDWELL   1802
PRRC2B[Homo_sapiens(human)]   LPVPPIEFGVSPKDSDFSLPPG--SASGPTGSPVVKLQDALASNAGLTQSIPILRRDHHI   1836
PRRC2C[Homo_sapiens(human)]   ISVSSAEYGTNAKESVTDYTTPSSSLPNTVATNNTKMEDTLVNNVPLPNTLPLPKRE-TI   2230
                                    ::*.. *:*         .                  .. : :::*: :    :

PRRC2A[Homo_sapiens(human)]   PSA-------AASA------EPQSKNLDSGHCVPEPSSS---GQRLYPEVFYG------S   1840
PRRC2B[Homo_sapiens(human)]   QRAIGLSPMSFPTADLTLKMESARKAWENSPSLPEQSSPGGAGSGIQPPSSVG------A   1890
PRRC2C[Homo_sapiens(human)]   QQSSSLTSVPPTTFSLTFKMESARKAWENSPNVREKGSPVTS---TAPPIATGVSSSASG   2287
                                :         :       *   *  :..  : * .*         *    *      .

PRRC2A[Homo_sapiens(human)]   -AGPSSSQISGGAMDSQ----LHP------------------------------------   1859
PRRC2B[Homo_sapiens(human)]   SSGVNYSSFGGVSMPPMPVASVAPSASMPGSHL---------------------------   1923
PRRC2C[Homo_sapiens(human)]   PSTANYNSFSSASMPQIPVASVTPTASLSGAGTYTTSSLSTKSTTTSDPPNICKVKPQQL   2347
                               :  . ..:.. :*       : *
```

```
PRRC2A[Homo_sapiens(human)]      ------------------------------------------------------------      1859
PRRC2B[Homo_sapiens(human)]      ----------------------------------------PPLYLDG-HVFA-SQPRLVP      1941
PRRC2C[Homo_sapiens(human)]      QTSSLPSASHFSQLSCMPSLIAQQQQNPQVYVSQSAAAQIPAFYMDTSHLFNTQHARLAP      2407

PRRC2A[Homo_sapiens(human)]      -----------------------------------NSGGFRPG---------T-PSLHPY      1874
PRRC2B[Homo_sapiens(human)]      QTIPQQQSYQ----QAAAAQQIPISLHTSLQA--QAQLGLRGGLPVSQSQEIF-SSLQPF      1994
PRRC2C[Homo_sapiens(human)]      PSLAQQQGFQPGLSQPTSVQQIPIPIYAPLQGQHQAQLSLGAGPAVSQAQELFSSSLQPY      2467
                                                                     . .:  *            **:*:

PRRC2A[Homo_sapiens(human)]      RSQPLYLPPGPAPPSA--LLSGLALKGQFLDFSTMQATELGKLPAGGVLYPPP-----SF      1927
PRRC2B[Homo_sapiens(human)]      RSQV-YMHPSLSPPSTMILSGGTALKPPYSAFPGMQPLEMVKPQSGS-------------      2040
PRRC2C[Homo_sapiens(human)]      RSQPAFMQSSLSQPSV--VLSGTAI----HNFPTVQHQELAKAQSGLAFQQTSNTQPIPI      2521
                                 ***  ::  . : **.  : .* *:      *  :*  *: *  :*

PRRC2A[Homo_sapiens(human)]      LYS------PAFCPSPLPDTSLLQVRQDLPSPSDFYSTPLQPG-GQSGFLPSGAPAQ---      1977
PRRC2B[Homo_sapiens(human)]      --------------------------------------PYQPMSGNQALVYEGQLSQAAG      2062
PRRC2C[Homo_sapiens(human)]      LYEHQLGQASGLGGSQLIDTHLLQARANLTQASNLYSGQVQQP-GQTNFYNTAQS-----      2575
                                                                         *   *:  :   .

PRRC2A[Homo_sapiens(human)]      QMLLPMVDSQLPVVN-----FGSLPPAPPPAPPPLSLLPVGPAL---------QPPSL--      2021
PRRC2B[Homo_sapiens(human)]      LGASQMLDSQLPQLTMPLPRYGSGQQ--PLILPQSIQLPPGQSL---------SVGAP--      2109
PRRC2C[Homo_sapiens(human)]      PSALQQVTVPLPASQLSLPNFGSTGQ--PLIALPQTLQPPLQHTTPQAQAQSLSRPAQVS      2633
                                       :   **        :**     *         *              .  :

PRRC2A[Homo_sapiens(human)]      ---AVRPPPAPATRVLPSPARPFPASLGRAELHPVELKPFQDYQK-LSSNLGGPGSSR--      2075
PRRC2B[Homo_sapiens(human)]      ---RRIPPPGSQPPVLNTS----------REPSQMEMKGFHFADSKQNVPSGGPVPSPQT      2156
PRRC2C[Homo_sapiens(human)]      QPFRGLIPAGTQHSMIA----------TTGKMSEMELKAFGSG---IDIKPGTPPIAGRS      2680
                                        * .    ::              :   :*:* *       .   * *  :

PRRC2A[Homo_sapiens(human)]      TPPTGRSFSGL-----NSRLKATPSTYSGVFRTQRVDLYQQASPPDALRWIPK-------      2123
PRRC2B[Homo_sapiens(human)]      YRPSSASPSGK-----PSGSAVNMGSVQGHYVQQAKQ-----------------------      2188
PRRC2C[Homo_sapiens(human)]      TTPTSSPFRATSTSPNSQSSKMNSIVYQKQFQSAPATVRM--TQPFPTQFAPQAKQRAEV      2738
                                   *:.    .       .    .    .  :

PRRC2A[Homo_sapiens(human)]      -------------------------------PWERTGPPPREGPSRRA----EEPGSR-G      2147
PRRC2B[Homo_sapiens(human)]      ----------------------------------RVDEKPSLGAVKLQ----EAPSAASQ      2210
PRRC2C[Homo_sapiens(human)]      LQSTQRFFSEQQQSKQIGGGKAQKVDSDSSKPPETLTDPPGVCQEKVEEKPPPAPSIATK      2798
                                                                        *     :        *.

PRRC2A[Homo_sapiens(human)]      DKEPGLPPPR---------      2157
PRRC2B[Homo_sapiens(human)]      MKRTGAIKPRAVKVEESKA      2229
PRRC2C[Homo_sapiens(human)]      PVRTGPIKPQAIKTEETKS      2817
                                   . *   *:
```

Supplemental Material and Methods

Reagents, antibodies, plasmids, and siRNAs

Cloning Primers (F, forward; R, reverse)	Product	Sequence (5' - 3')
human_PRRC2B_F	FLAG-tagged full-length PRRC2B, P1, T1, T2, T3, T4	CGGGGTACCGCCACCATGTCCGATCGTTTGGGGCA
human_PRRC2B_750_R	FLAG-tagged P1	ATAAGAATGCGGCCGCCACATGTAGCCCTCTGGGCTC
human_PRRC2B_150_R	FLAG-tagged T1	ATAAGAATGCGGCCGCCACTTTCCATTCAGCTGTGCCCAT
human_PRRC2B_300_R	FLAG-tagged T2	ATAAGAATGCGGCCGCCATCGTTCCACTGTACCCTGGTT
human_PRRC2B_450_R	FLAG-tagged T3	ATAAGAATGCGGCCGCCAGTCTGGCGCCTTTCGGAC
human_PRRC2B_600_R	FLAG-tagged T4	ATAAGAATGCGGCCGCCACTGTGCCACTGCTGGGGA
human_PRRC2B_750_F	FLAG-tagged P2	CGGGGTACCGCCACCATGGCACTGCAGAGCAAGGGC
human_PRRC2B_1500_R	FLAG-tagged P2	ATAAGAATGCGGCCGCCAACTGGCATGGGCTGCCCGC
human_PRRC2B_1500_F	FLAG-tagged P3	CGGGGTACCGCCACCATGGCTGACCTTCCCGAAGCCTCC
human_PRRC2B_2230_R	FLAG-tagged P3, full-length	ATAAGAATGCGGCCGCCAGGCCTTACTCTCCTCCACTTTGACAG
CCND2_UTR5_F	WT-FLuc	AAGCTAATACGACTCACTATAGGCAGCCCCGAGGCTCTG
CCND2_UTR5_R	WT-FLuc	CCTTTGGCTAAATAGGGGGTTTT
CCND2_UTR5_mutant_R	Del-FLuc, T2A-FLuc, C2G-FLuc, CT2GA-FLuc	GCCTCGGGGCTGCCTC
CCND2_UTR5_T2A_F	T2A-FLuc	ACAGCACGCCCACCACCCAAACCACGCCACCCAACAGCACCACCAACACACACAGCCCACACCACACCCCCG AAAACCCCCTATTTAGCCAAAGG

CCND2_UTR5_C2G_F	C2G-FLuc	TGTGTGGGGGAGGAGGGAATGGTGGGGTGGGTTGTGGTGGAGGTTGTGTGTGTGGGGTGAGGTGTGGGGGGAAAACCCCCTATTTAGCCAAAGG
CCND2_UTR5_Del_F	Del-FLuc	AAGGAGGTCAGGGGAACGCTCTCCCCTCCCCTTCCAAAAAACAAAAACAGAAAAACCTTTTTCCAGGCCGGGAAAACCCCCTATTTAGCCAAAGG
CCND2_UTR5_CT2GA_F	CT2GA-Fluc	AGAGGAGGGGGAGGAGGGAAAGGAGGGGAGGGAAGAGGAGGAGGAAGAGAGAGAGGGGAGAGGAGAGGGGGGAAAACCCCCTATTTAGCCAAAGG
**RT-qPCR Primers (F, forward; R, reverse)**	**Targeted transcript (RefSeq)**	**Sequence (5’ - 3’)**
CCND2_F	NM_001759.4	CTCTGCTGAGCGGTACTAAAC
CCND2_R	NM_001759.4	CTCCCTTCAACTATCATCCCATAC
CRKL_F	NM_005207.4	CACACGGAAAGCATGGAAATAG
CRKL_R	NM_005207.4	AAACTGCAGGTAGAGGAGTTG
YWHAZ_F	NM_145690.3	AGCAGAGAGCAAAGTCTTCTATT
YWHAZ_R	NM_145690.3	GACTGATCGACAATCCCTTTCT
GAPDH_F	NM_002046.7	GACCACTTTGTCAAGCTCATTTC
GAPDH_R	NM_002046.7	CTCTCTTCCTCTTGTGCTCTTG
GATA4_F	NM_002052.5	TTCCAGCAACTCCAGCAACG
GATA4_R	NM_002052.5	GCTGCTGTGCCCGTAGTGAG

ACTB_F	NM_001101.5	GGATCAGCAAGCAGGAGTATG
ACTB_R	NM_001101.5	AGAAAGGGTGTAACGCAACTAA
CTC1_F	NM_025099.6	CTCTTCTGGGTGTGTGCTTATT
CTC1_R	NM_025099.6	CTGATGATGGCCTGGCTTATAG
ATAD5_F	NM_024857.5	AGCTGATCCTGTCCCTAGTT
ATAD5_R	NM_024857.5	TAGGAAGTCACGCTTTGCTTTA
SERBP1_F	NM_015640.4	GTGACTGAGGAAACACCTGAA
SERBP1_R	NM_015640.4	AGCCTTCCACTCATCCAAAG
BPTF_F	NM_182641.4	TTATCCGGAGGAGATGGAAGA
BPTF_R	NM_182641.4	GAGTGCTGCTGTAGGTACTATG
PPP2R1A_F	NM_014225.6	ATGGGTCTCTCTCCCATCTT
PPP2R1A_R	NM_014225.6	GTTCACACAGTCCAGGTTAGAG
RPL35_F	NM_007209.4	GGAGAACCTGAAGACCAAGAAG
RPL35_R	NM_007209.4	CTCAGCCAGCTGTGCTTTAT
RPLP1_F	NM_001003.3	GCTGTTGGTCTTGTCCATAGT
RPLP1_R	NM_001003.3	GCCTGTGTCATGGCAGAATA
RPL38_F	NM_000999.4	GGGAGGCCATGATGGAAATA
RPL38_R	NM_000999.4	GCGAGTGGTAGATGTGCTATAC

PTBP1_F	NM_002819.5	GACGGCATTGTCCCAGATATAG
PTBP1_R	NM_002819.5	GGTCCGTTAGTGACACAAGTAG
HSPA4_F	NM_002154.4	CATTGCAGTGTGCCATCTTATC
HSPA4_R	NM_002154.4	CACAGTCACTTGACCCTTCTT
RPL37A_F	NM_000998.5	ATTACTTGAGGCCAGGAGATTG
RPL37A_R	NM_000998.5	CCTCCAAAGTAGCTGGGATTAC
**ASO**	**Effect**	**Sequence (5’ - 3’)**
ctrl ASO	control ASO with no target in eukaryotic cells	CmCmAmGmGmAmUmUmCmAmAmCmCmUmAmoCm
ASO1	ASO targeting PRRC2B-binding sites on 5' UTR of *CCND2* mRNA	UmUmAmGmGmAmGmCmGmGmAmGmGmGmAmAmGmAm
ASO2	ASO targeting an adjacent region of PRRC2B-binding sites on 5' UTR of *CCND2* mRNA	CmCmUmCmCmUmUmCmCmUmUmUmGmGmCmUm
Note: “m” indicates a 2'-O-methyl modification.		
**Antibodies**	**Cat. #**	**Dilution**
Mouse anti-β-actin	MA5-11869; Invitrogen	IB (1: 2000)
Mouse anti-DAP5 (EIF4G2)	135999; Santa Cruz	IB (1: 2000)
Donkey anti-rabbit IgG	NA9340V; Roche	IP (1: 100)
Rabbit anti-eIF3G Polyclonal antibody	11165-1-AP; Proteintech	IB (1:1000)
Mouse monoclonal anti-eIF3D	sc-271515; Santa Cruz	IB (1:500)
Mouse monoclonal anti-eIF4G1	sc-133155; Santa Cruz	IB (1:500)

Mouse monoclonal anti-eIF4E	sc-9976; Santa Cruz	IB (1:500)
Mouse monoclonal anti-FXR1	sc-137418; Santa Cruz	IB (1:500)
Rabbit polyclonal anti-PRRC2B	PA5-66677; Thermo Fisher Scientific	IB (1:1000); IP (1:125)
Mouse polyclonal anti-RPLP0	sc-293260; Santa Cruz	IB (1:500)
Rabbit polyclonal anti-RPS6	14823-1-AP; Proteintech	IB (1:1000)
Pierce™ Anti-DYKDDDDK Magnetic Agarose	A36797; Thermo Fisher Scientific	IP (1:50)
Mouse monoclonal anti-CRKL	sc-365092; Santa Cruz	IB (1:500)
Rabbit polyclonal anti-YWHAZ	A7639; Abclonal	IB (1:1000)
Rabbit polyclonal anti-cyclin D2	A1773; Abclonal	IB (1:1000)
**Cell-lines**	**Cat. #**	
HEK293T	CRL-3216; ATCC	
**Chemicals & reagents**	**Cat. #**	
Ethanol	V1016; Decon Labs	
Chloroform	C2432-500ML; Millipore-Sigma	
RIPA Lysis and Extraction Buffer	89900; Thermo Fisher Scientific	
[gamma-32P]-ATP (250 mCi)	NEG502A250UC; PerkinElmer	
HEPES-KOH	BB-2076-K; Boston BioProducts	
Potassium chloride	P3911-25G; Millipore-Sigma	

$MgCl_2$	M8266-100G; Millipore-Sigma	
Sodium Acetate 3M, pH 5.2	41920020; Bio-world	
Dithiothreitol	D0632-10G; Millipore-Sigma	
IGEPAL® CA-630	I8896-50ML; Millipore-Sigma	
Vanadyl ribonucleoside complexes	94740-250MG; Millipore-Sigma	
RNasin® Ribonuclease Inhibitor	N2611; Promega	
iScript™ cDNA Synthesis Kit	1708890; Bio-Rad	
cOmplete™ Protease Inhibitor Cocktail	11697498001; Millipore-Sigma	
iTaq Universal SYBR Green Supermix	1725124, Bio-Rad	
Picrosirius red stain	ab150681; Abcam	
TrueCut Cas9	A36496; Thermo Fisher Scientific	
Trypsin-EDTA (0.25%), phenol red	25200056; Thermo Fisher Scientific	
6-well cell culture plates	140675; Thermo Fisher	
10-cm cell culture plates	353002; Corning	
OPTI-MEM	31985062; Thermo Fisher Scientific	
Immobilon®-P PVDF Membrane	IPVH00010; Millipore-Sigma	
Lipofectamine 3000	L3000015; Thermo Fisher Scientific	
Superscript III	18080093; Thermo Fisher Scientific	

Dual-Glo Luciferase Assay System	E2920; Promega	
Bsmb1 restriction enzyme	R0739S; NEB	
Kpn1 restriction enzyme	R3142S; NEB	
Not1 restriction enzyme	R3189S; NEB	
Quick CIP	M0525S; NEB	
RNase T1 (1000 U/μL)	EN0542; Thermo Fisher Scientific	
T4 DNA Ligase	M0202S; NEB	
T4 PNK	M0201S; NEB	
Dpn1 restriction enzyme	R0176S; NEB	
Proteinase K	3115879001; Millipore Sigma	
GlycoBlue™ Coprecipitant (15 mg/mL)	AM9515; Thermo Fisher Scientific	
Acid-Phenol: Chloroform, pH 4.5 (with IAA, 125:24:1)	AM9722; Thermo Fisher Scientific	
RNA Gel Loading Dye (2X)	R0641; Thermo Fisher Scientific	
NuPage transfer buffer 20X	NP00061; Thermo Fisher Scientific	
UltraPure Phenol:Chloroform: Isoamyl Alcohol (25:24:1, v/v)	15593031; Thermo Fisher	
Trizol	15596026; Thermo Fisher	
TRIPZ Inducible Lentiviral shRNA targeting PRRC2B	V3THS_374329; Dharmacon	

4-Thiouridine	T4509; Sigma-Aldrich	
Cycloheximide	C7698; Sigma-Aldrich	
siRNA smartpool (eIF4G2)	L-011263-00-0005; Dharmaco	
**Plasmids**	**Cat. #**	
pGL3-TK-5UTR-BsmBI-Luciferase	114670; Addgene	
pRL Renilla Luciferase Control Reporter	E2231; Promega	
pCDNA3.1(+)-3xFLAG	Homemade	

MIQE for RT-qPCR

For all RT-qPCR experiments in this study, > $1x10^9$ HET293T cells transfected with shRNAs, antisense oligos (ASOs), or luciferase reporters were used as experimental and control groups. All cell samples were obtained from cultured cells without dissection. If not used immediately, samples were snap-frozen in liquid nitrogen and stored at -80°C for up to 12 months.

For RNA extraction, cells were lysed with 1000 μl of Trizol (Qiagen) and mixed with 200 μl chloroform. The mixture was spun down at 16,000 g for 10 min. RNA was precipitated from the aqueous layer by adding two volumes of isopropanol and spinning down at 16,000 g for 10 min. The pellet was washed twice with 70% ethanol, left to dry, and resuspended in nuclease-free water. Potential genomic DNA contamination was removed by incubating with DNase I at 37°C for 10 min, followed by another round of RNA purification using chloroform: phenol: isoamyl alcohol, similar to those mentioned in the method section. Purified RNA was subject to 1% agarose gel electrophoresis to check for RNA integrity (reflected by 28S/18S > 1.0) and genomic DNA contamination. RNA quantification was performed by measuring A260 using Nanodrop (Thermofisher ND-ONE-W). RNA purity was assessed by A260/A280. Inhibition of reverse transcription was

tested using 1000 ng, 500 ng, and 250 ng of RNA. Cq values of the inhibition test are shown in **Table S8**.

cDNAs were prepared using iScript master mix RT Kit (Bio-Rad) following the manufacturer's suggestions. Briefly, 500-1000 ng of RNA were added to a 20 μl reaction containing reverse transcriptase, RNase inhibitor, dNTPs, primers (optimum blend of oligo(dT) and random primers), $MgCl_2$, and stabilizers. Reactions were performed at the following conditions: (priming, 5 min at 25°C; reverse transcription, 20 min at 46°C; RT inactivation, 1 min at 95°C). No-RT control was included to ensure the synthesis of cDNA.

All qPCR primers were designed by the IDT qPCR primer design tool with no multiplex and synthesized as single-stranded DNAs by IDT with no extra modification. All primers target the exons in the coding region of the main open reading frame of the longest mRNA transcript (isoform) of each gene of interest. Detailed primer information and target sequence accession number are listed in Supplemental Material and Methods. All PCR amplicons are less than 200 nt long.

RT-qPCR-amplification was performed using SYBR Primer Assay kits (Bio-Rad 3 1752124) following the manufacturer's suggestions. 10 μl reaction was set up in Bio-Rad Hard-Shell® 96-Well PCR Plates (#HSP9601) with 1 μl primer (10 μM), 5 μl Bio-Rad iTaq Universal SYBR Green Supermix (containing an optimal amount of Mg^{2+}, hot-start iTaq DNA polymerase, and dNTP), 0.2 μl of cDNA from a 20 μl RT reaction, and RNase-free water. qPCR was performed using Bio-Rad CFX Connect Real-Time PCR Detection System with the following program: 1 min at 95°C, (10 s at 95°C, 40 s at 60°C, read plate) x 40 cycles, 10 s at 95°C, melt Curve 65 – 95°C, increment 0.5°C for 5 s, plate Read.

Cq values were determined by the regression mode in Bio-Rad CFX Connect Real-Time PCR Detection System. Cq values < 40 (no-template control has Cq value > 45) are considered reasonable. For each primer set, the identity of the resulting PCR product was confirmed by cloning and Sanger sequencing. Melting curves were used in each subsequent PCR to verify that each primer set reproducibly and specifically generates the same PCR product. A standard curve for each primer set was generated using 1x, 2x, 2.5x, 4x, 8x, 16x, 25x, 250x, 2500x, and 25000x diluted RNA (**Table S8**). Linear models were fitted using Microsoft Excel to calculate slope, y-intercept, and PCR efficiency in

order to determine the Linear range of detection. At least biological duplicates and technical duplicates were performed for each measurement. Measurements with Cq variant (intra-assay) greater than 1 were repeated.

For data analysis, relative mRNA abundance was calculated by the Livak-Schmittgen method {2}^{-\Delta \Delta Cq}. Normalization was performed against 18S rRNA (internal control) based on its constant high expression in all samples or Renilla mRNA spike-in (if used). All quantitative data were presented as mean ± SD and analyzed using Excel (Microsoft Office). Statistical analyses were performed using the two-tailed Student's *t* test with $P < 0.05$ considered significant.
